# Searching for Novel Sources of Hydrogen Sulfide Donors: Chemical Profiling of *Polycarpa aurata* Extract and Evaluation of the Anti-Inflammatory Effects

**DOI:** 10.3390/md21120641

**Published:** 2023-12-15

**Authors:** Marcello Casertano, Erika Esposito, Ivana Bello, Chiara Indolfi, Masteria Yunovilsa Putra, Lorenzo Di Cesare Mannelli, Carla Ghelardini, Marialuisa Menna, Raffaella Sorrentino, Giuseppe Cirino, Roberta d’Emmanuele di Villa Bianca, Concetta Imperatore, Elisabetta Panza, Emma Mitidieri

**Affiliations:** 1 Department of Pharmacy, School of Medicine and Surgery, University of Naples Federico II, Via D. Montesano 49, 80131 Naples, Italy; marcello.casertano@unina.it (M.C.); erika8esposito@gmail.com (E.E.); iv.bello.ib@gmail.com (I.B.); mlmenna@unina.it (M.M.); rafsorre@unina.it (R.S.); cirino@unina.it (G.C.); e.panza@unina.it (E.P.); emma.mitidieri@unina.it (E.M.); 2 Department of Molecular Medicine and Medical Biotechnology, School of Medicine and Surgery, University of Naples Federico II, 80131 Naples, Italy; chiara.indolfi@unina.it; 3 Research Center for Vaccine and Drugs, Research Organisation for Healths, National Research and Innovation Agency (BRIN), Jalan Raya Jakarta-Bogor KM. 46, Cibinong 16911, Indonesia; masteria.yunovilsa.putra@brin.go.id; 4 Department of Neuroscience, Psychology, Drug Research and Child Health-NEUROFARBA-Pharmacology and Toxicology Section, University of Florence, 50139 Florence, Italy; lorenzo.mannelli@unifi.it (L.D.C.M.); carla.ghelardini@unifi.it (C.G.)

**Keywords:** marine natural products, *Polycarpa aurata*, tunicates, secondary metabolites, LC-HRMS, H_2_S-releasing agents, inflammation, edema, mice

## Abstract

Hydrogen sulfide (H_2_S) is a signaling molecule endogenously produced within mammals’ cells that plays an important role in inflammation, exerting anti-inflammatory effects. In this view, the research has shown a growing interest in identifying natural H_2_S donors. Herein, for the first time, the potential of marine extract as a source of H_2_S-releasing agents has been explored. Different fractions obtained by the Indonesian ascidian *Polycarpa aurata* were evaluated for their ability to release H_2_S in solution. The main components of the most active fraction were then characterized by liquid chromatography–high-resolution mass spectrometry (LC-HRMS) and NMR spectroscopy. The ability of this fraction to release H_2_S was evaluated in a cell-free assay and J774 macrophages by a fluorimetric method, and its anti-inflammatory activity was evaluated in vitro and in vivo by using carrageenan-induced mouse paw edema. The anti-inflammatory effects were assessed by inhibiting the expression of inducible nitric oxide synthase (iNOS), cyclooxygenase-2 (COX2), and interleukin-6 (IL-6), coupled with a reduction in nitric oxide (NO) and IL-6 levels. Thus, this study defines the first example of a marine source able to inhibit inflammatory responses in vivo through the release of H_2_S.

## 1. Introduction

In the years leading up to the mid-2000s, most of the scientific literature focused on hydrogen sulfide (H_2_S) primarily revolved around toxicological investigations. However, as time progressed, a shift in understanding occurred [1]. H_2_S has begun to be acknowledged as a signaling molecule endogenously produced within mammals’ cells and tissues. This recognition placed H_2_S in the same category as other molecules referred to as “gasotransmitters,” which includes nitric oxide (NO) and carbon monoxide (CO) [2]. These molecules share numerous characteristics and, in many instances within biological systems, work together in a synchronized and collaborative manner [3,4,5,6].

H_2_S is endogenously produced by three enzymes—cystathionine-β-synthase (CBS), cystathionine-γ-lyase (CSE), and 3-mercapto pyruvate sulfurtransferase (3-MST)—as well as by a non-enzymatic reaction [7,8,9,10]. Like NO and CO, H_2_S may reach both aqueous and hydrophobic milieu due to its small size and slight polarity. Hence, it is involved in the regulation of various biological systems in both physiological and pathological conditions [11,12,13,14].

The role of H_2_S in inflammation is widely described in the literature [15,16,17]. However, whether this gas exerts a pro- or anti-inflammatory effect or contributes to the resolution of inflammation remains unclear [17]. The role of H_2_S as a pro- or anti-inflammatory mediator is influenced by various factors, including the type of animal models used, the nature of inflammation (acute or chronic), and, importantly, whether the H_2_S source originated within the organism (endogenous) or was introduced externally (exogenous, i.e., donor-derived) [18,19,20,21,22]. Therefore, the choice of an H_2_S donor becomes crucial. The release mechanism and kinetics from each donor can make a difference in terms of the final results, especially in terms of the bioavailability of the desired sulfur species. Two categories of H_2_S donors, classified as “fast” or “slow” releasing donors, are employed in both in vitro and in vivo models of inflammation. For instance, inorganic salts like NaHS and Na_2_S quickly release H_2_S in short-lived bursts, creating high concentrations of H_2_S within cells. However, this mechanism does not replicate the steady physiological production of H_2_S by the body’s enzymes [23]. To address the limitations posed by inorganic sulfide salts, a growing interest surrounds H_2_S donors with controlled release rates. For example, slow donors like GYY4137, AP 39, and AP123 have demonstrated beneficial effects in various models of inflammatory diseases. GYY4137, for instance, has been shown to improve intestinal epithelial barrier integrity and alleviate DSS (dextran sodium sulfate)-induced inflammation in vivo, as well as mitigating myocardial injury in cases of sepsis-induced cardiomyopathy [24,25]. Research by Whiteman and colleagues in 2010 revealed that GYY4137 could reduce the synthesis of pro-inflammatory cytokines in mouse macrophages challenged with *Escherichia coli* lipopolysaccharide (LPS) [26]. Furthermore, the unique triphenylphosphonic moiety found in AP39 and AP123 facilitates the drug’s accumulation in mitochondria in a potential-dependent manner. This ensures that the concentration of H_2_S remains within a safe range, as the normalization of mitochondrial potential prevents excessive drug accumulation. Additionally, these compounds have exhibited the ability to protect against prolonged low-level oxidative stress induced by hyperglycemia in endothelial cells [27]. In addition to the above-mentioned H_2_S donors, several small-molecule donors capable of releasing H_2_S under various conditions have been identified. In this view, carbonyl sulfide (COS) represents an intermediate that can be rapidly converted to H_2_S by the ubiquitous mammalian enzyme carbonic anhydrase. This approach has the advantage that it can be easily tuned to specific activation groups and enzymes [28].

In addition, a good number of sustained-release persulfide drugs have been developed in recent years. Indeed, considering the relevance of S-persulfidation and glutathionylation in cell signaling, several persulfide and glutathione persulfide donors have been designed and examined for their antioxidant effect, taking into account a better protective effect than the corresponding thiols [29]. In parallel, alternative approaches rely on clinically approved drugs of various classes (e.g., nonsteroidal anti-inflammatories) coupled with H_2_S-donating groups. In this scenario, ATB-346, an H_2_S-donating derivative of the non-steroidal anti-inflammatory compound naproxen, is the most advanced compound in clinical trials. Interestingly, a growing body of evidence reports that ATB-346 is more effective than naproxen in terms of anti-inflammatory properties combined with improvement in the status of gastric mucosa [30,31].

In this context, natural H_2_S donors existing in plants have shown remarkable biological activity. To date, there is a large body of literature showing the beneficial effect of natural products as H_2_S releasers, such as glucoerucin, glucoraphanin, diallyl trisulfide (DATS), and diallyl disulfide (DADS) occurring in rocket, cruciferous vegetables, or garlic [32,33,34,35,36,37]. Alongside these compounds, many other small natural molecule polysulfides have received attention as equally or more active sources of H_2_S or alternatively viable reservoirs of sulfane sulfur through the transsulfuration pathway [38].

Over the last two decades, several researchers have investigated different combinations of phytochemicals and plant extracts on outcomes of disease prevention or treatment since the use of natural product mixtures is often more effective than purified compounds due to beneficial “synergistic” interactions [39]. The approach of bioassay-guided fractionation, historically effective in identifying active compounds, frequently encounters a drawback of the loss of activity during the fractionation process. Due to this issue, it’s common to take initial steps for structural assessment, aiming to identify samples that contain both known and unknown active components. This process is referred to as “dereplication,” and it is intended to prioritize samples that are likely to include novel and biologically significant substances. This prioritization enhances the efficient utilization of natural resources in the pursuit of discovering new lead compounds. Dereplication involves comparing the characteristic spectral patterns of various constituents within a mixture. This comparison is typically carried out using techniques such as mass spectrometry, nuclear magnetic resonance (NMR), or UV spectroscopy. The goal is to search for compounds with known structures possessing matching spectral fingerprints in a dedicated dereplication database.

In recent years, the less-explored marine world has increasingly gained researcher’s attention thanks to technological advances that have made it possible to investigate the biodiversity of this environment. Marine natural products (MNPs) are considered a potentially important source of new drug leads [40,41,42,43,44,45,46,47] since they are structurally distinct from terrestrial ones due to the different environments in which they evolved. Nevertheless, no data are available in the literature regarding MNPs and/or marine extracts as a source of H_2_S donors. Here, for the first time, we have analyzed the potential of the fractionated extract from the Indonesian solitary ascidian *Polycarpa aurata* (Quoi and Gaimard, 1834) as a source of H_2_S-releasing agents. This marine organism, a common constituent of the benthic invertebrate community of Indo-Pacific coral reefs, has been selected since it has already proved to be an extremely rich source of unique alkaloid-type structures with rare sulfur-containing functional groups [48,49,50]. In this study, we first assessed the ability of the different fractions to release H_2_S in solution and characterized the main components of the most active fraction using liquid chromatography coupled with high-resolution mass spectrometry (LC-HRMS) and NMR spectroscopy. Moreover, we have defined its anti-inflammatory effect in in vitro and in vivo models, identifying the first fraction, obtained from a marine natural extract, as a source of H_2_S.

## 2. Results

### 2.1. Extraction and Fractionation of P. aurata

The solitary ascidian *P. aurata*, collected from the Indonesian coast, was exhaustively extracted with methanol and, subsequently, with chloroform. The combined extracts were concentrated in vacuo, and the resulting residue was partitioned between water and butanol. The butanol soluble portion was subjected to chromatography over a reversed-phase silica gel column eluting with a solvent gradient from H_2_O/MeOH 9:1 (*v*/*v*) to MeOH 100%. Five fractions (PAB1-PAB5) were obtained, and all were analyzed to assess their ability to release H_2_S in solution.

### 2.2. PAB1-PAB5 Release H_2_S

**Figure 1:**
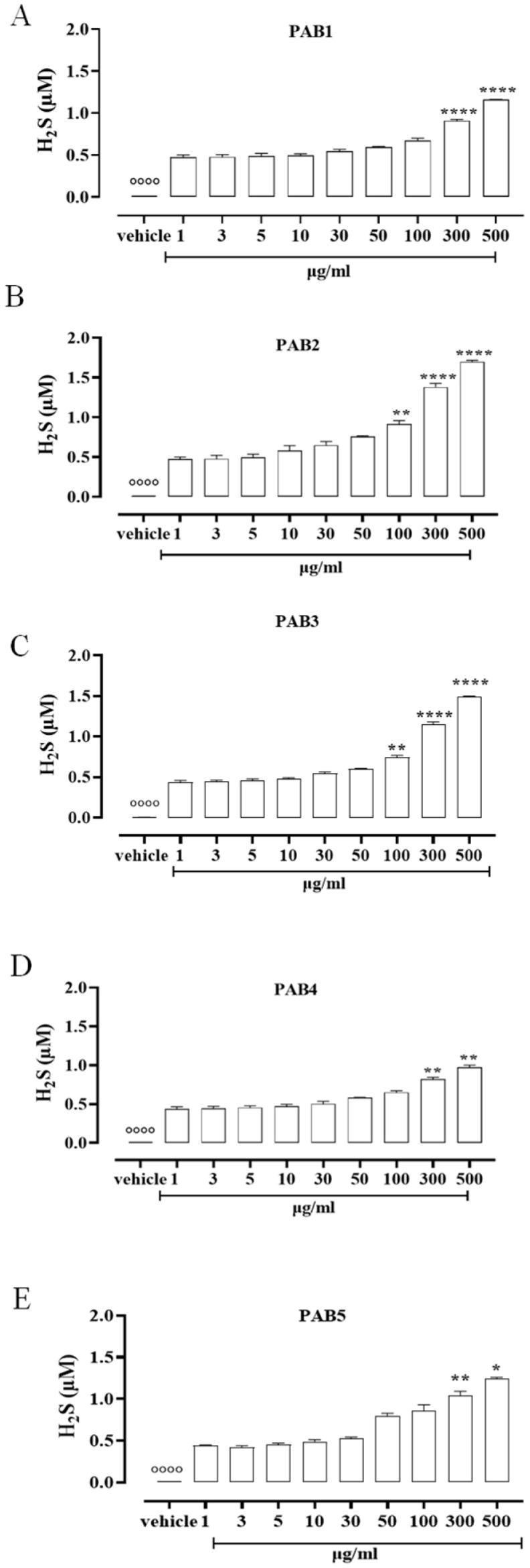
H_2_S measurement. (**A**) PAB1, (**B**) PAB2, (**C**) PAB3, (**D**) PAB4, and (**E**) PAB5 (1–500 µg/mL) release H_2_S in a cell-free assay. Data are reported as µM and expressed as means ± SEM (*n* = 3). °°°° *p* < 0.0001 vs. all concentrations; * *p* < 0.05, ** *p* < 0.01, and **** *p* < 0.0001 vs. preceding concentrations.

To assess the ability of the five fractions obtained by the Indonesian *P. aurata* to release H_2_S in solution, a fluorescence method based on the selective sulfide detector SF7-AM was achieved. Data obtained by cell-free assay show that all the fractions, PAB1, PAB2, PAB3, PAB4, and PAB5, released H_2_S with similar profiles but different strengths compared to the vehicle (°°°° *p* < 0.0001 vs. all concentrations, Figure 1). At lower concentrations, all the fractions tested released a similar amount of H_2_S (Figure 1). At growing concentrations of PAB1, PAB2, PAB3, PAB4, and PAB5, a significant increase in H_2_S release was found (Figure 1, * *p* < 0.05, ** *p* < 0.01 and **** *p* < 0.0001 vs. preceding concentrations). Interestingly, only PAB2 and PAB3 showed a significant increase in H_2_S release already at 100 µg/mL (Figure 1B,C ** *p* < 0.01). However, the amount of H_2_S obtained from PAB2 100 µg/mL was higher than from PAB3 at the same concentration (0.91 ± 0.04 vs. 0.75 ± 0.02; * *p* < 0.05), 300 µg/mL (1.38 ± 0.045 vs. 1.146 ± 0.035; * *p* < 0.05), and 500 µg/mL (1.7 ± 0.017 vs. 1.495 ± 0.006; *** *p* < 0.001). Based on this evidence, among the five fractions evaluated, PAB2 is the most effective for releasing H_2_S, and thus it was selected for further investigation.

**Figure 2:**
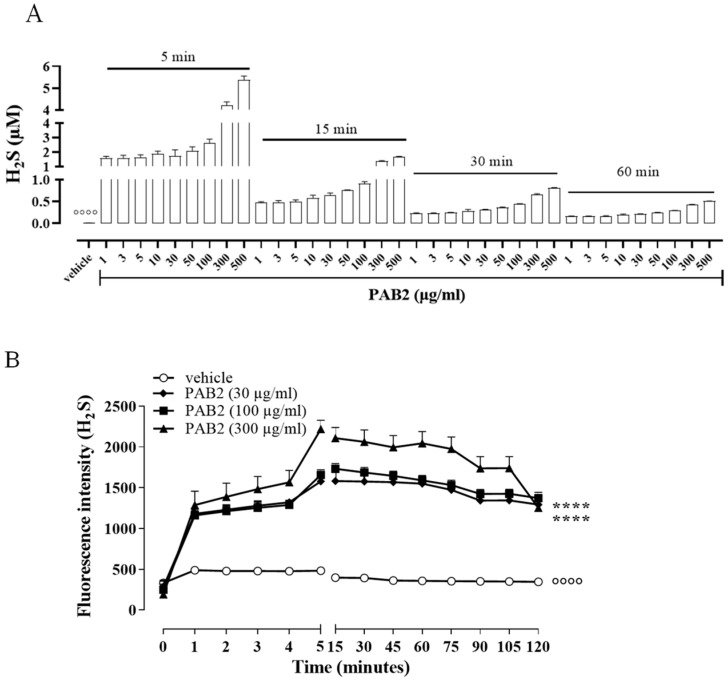
Kinetic of H_2_S release. (**A**) Measurement of H_2_S release by PAB2 up to 60 min (1–500 µg/mL) in a cell-free assay. Data are reported as µM and expressed as means ± SEM (*n* = 3; °°°° *p* < 0.0001 vs. all concentrations for each time point). (**B**) Live fluorometric quantification of H_2_S in J774 exposed to PAB2 (30–300 µg/mL). Data are reported as fluorescence intensity and expressed as means ± SEM (*n* = 3; °°°° *p* < 0.0001 vs. PAB2 30, 100 and 300 µg/mL; **** *p* < 0.0001 vs. PAB2 300 µg/mL).

In order to evaluate the kinetic for H_2_S formation, the release of H_2_S was monitored for up to 60 min. Of note, in this experimental condition, i.e., in a cell-free assay, the amount of H_2_S released from PAB2 is at its maximum at 5 min and declines at 60 min (Figure 2A). However, H_2_S released by PAB2 was significantly higher compared to the vehicle at all concentrations for each time point (Figure 2A; °°°° *p* < 0.0001).

In another setting of experiments, the release of H_2_S was evaluated in J774 cells following the exposure of PAB2 (30, 100, and 300 µg/mL). Intriguingly, PAB2 provoked a significant increase in the intracellular amount of H_2_S in J774 cells compared to the vehicle at all concentrations tested (Figure 2B; °°°° *p* < 0.0001). In detail, the lower concentrations tested (30 and 100 µg/mL) showed an overlapping profile, while the PAB2 fraction at 300 µg/mL reached the maximum effect at 5 min and persisted for up to 2 h, resulting in significantly higher values compared to 30 and 100 µg/mL (Figure 2B; **** *p* < 0.0001).

### 2.3. LC-HRMS and NMR Analyses of PAB2

The metabolic profiling of the most active fraction PAB2 (18.3 mg), eluted with H_2_O/MeOH 2:8 (*v*/*v*), was performed by liquid chromatography–high-resolution mass spectrometry (LC-HRMS, Figures S1–S8) and NMR spectroscopy, both 1D and 2D (Figures S9–S14), in order to identify its chemical composition.

**Figure 3:**
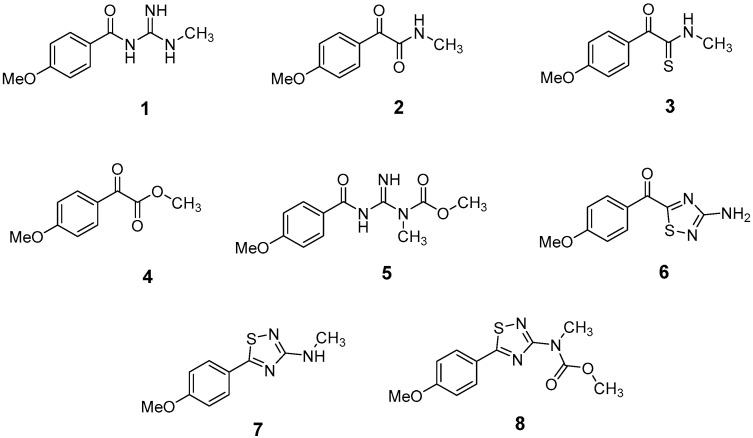
Structures of the identified secondary metabolites **1**–**8** from PAB2 fraction of *P. aurata* BuOH extract.

The LC-HRMS^n^ (*n* = 1, 2) method for PAB2 analysis was implemented by using a reverse phase column. The mobile phases were water (eluent A) and methanol (eluent B), both containing 0.1% formic acid. In detail, the gradient elution at a flow rate of 0.5 mL/min was 5 to 95% B in 35 min, holding the elution H_2_O/MeOH 5:95 for 5 min. Compounds **1**–**8** (Figure 3) were tentatively identified in the PAB2 fraction obtained from *P. aurata* BuOH extract based on the extensive analysis of both mass and NMR data compared with the available literature. The dereplicated compounds include *N*-(4-methoxybenzoyl)-*N’*-methylguanidine (**1**) [51], 2-(4-methoxyphenyl)-*N*-methyl-2-oxoacetamide (**2**) [51], 2-(4-methoxyphenyl)- *N*-methyl-2-oxothioacetamide (**3**) [50], methyl 2-(4-methoxyphenyl)-2-oxoacetate (**4**) [51], polyaurine A (**5**) [48], polycarpathiamine B (**6**) [49], and polyaurine B (**8**), along with its chemical degradation product (**7**) [48,52] (Figure 3).

**Figure 4:**
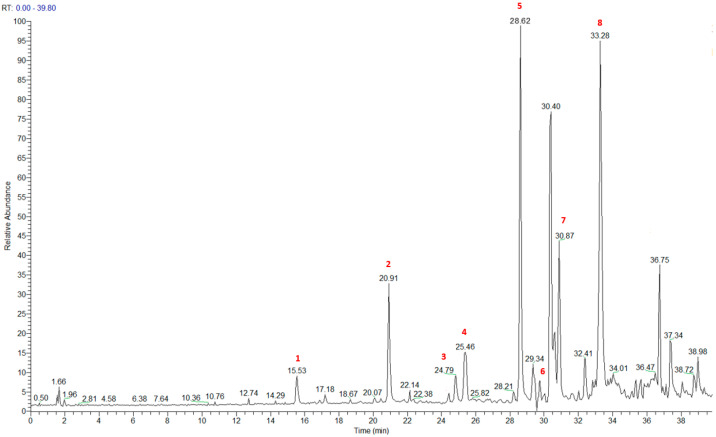
Total ion chromatogram of fraction PAB2 with related labels (red) for the identified compounds.

The HRMS and HRMS^2^ experiments allowed the assessment of the above structure by characterizing the main fragmentation pattern of compounds **1**–**8** putatively identified in PAB2; the obtained data have been summarized in Table 1 according to the chromatographic separation shown in Figure 4.

Compound **1** (RT = 15.53 min) was tentatively assigned as *N*-(4-methoxybenzoyl)-*N’*-methylguanidine. The HR full MS spectrum of **1** showed a protonated molecular ion peak at *m*/*z* 208.1078 [M + H]^+^, whereas the HRMS^2^ spectrum showed a fragment ion at *m*/*z* 191.0816, consistent with the loss of NH_3_, and at *m*/*z* 152.0705, related to the loss of the *N*-methylguanidine moiety (Figure S1). Moreover, the homonuclear ^1^H-^1^H COSY connectivity between the aromatic protons at *δ*_H_ = 7.01 and *δ*_H_ = 8.31 and the ^1^H-^13^C HMBC correlation between the latter proton with the carbonyl group at *δ*_C_ = 168.5 as well as the HMBC cross peak of *N*-CH_3_ group (*δ*_H_ = 3.31) with the carbon at *δ*_C_ = 156.3 are data strictly in agreement with the available literature, confirming the assigned structure for **1** (Figures S10, S11, S13, and S14) [51]. The mass spectral data of the HR-MS peak at *m*/*z* 194.0809 (RT = 20.91 min) indicated that compound **2** has a molecular formula C_10_H_11_NO_3_, and it also contains the *p*-methoxylbenzoyl fragment, as suggested by the base peak *m*/*z* 135.0 of its HR-MS/MS spectrum (Figure S2). Moreover, the observing ^3^*J*_H,C_ correlation (Figure S14) between the NH-CH_3_ at *δ*_H_ = 2.96 (s, 3H) and a further carbonyl group (*δ*_C_ = 163.0) assigned the remaining atoms that can be combined in one way, agreeing with the referenced structure **2** [51]. Similarly, the protonated molecular ion peak at *m*/*z* 195.0650 [M + H]^+^ (RT = 25.46 min) indicating the molecular formula C_10_H_10_O_4_ and the same mass fragmentation pattern shown for **2**, along with the recorded NMR data (Figures S4, S10–S14), allowed a tentative definition of compound **4** as methyl 2-(4-methoxyphenyl)-2-oxoacetate [51].

Compound **3** (RT = 24.79) has been tentatively assigned as *N*-methyl-2-(4-methoxyphenyl)-2-oxothioacetamide according to spectroscopic data already reported in the literature [50]. Indeed, the HRMS spectrum showed a protonated molecular ion at *m*/*z* 210.0584, attributable to the molecular formula C_10_H_11_NSO_2._ In addition, in this case, the base peak at *m*/*z* 135.0 agrees with the presence of a *p*-methoxylbenzoyl fragment (Figure S3). Further analysis of the HMBC and HSQC cross peaks of PAB2 fraction revealed the methine groups of the aromatic ring [*δ*_H_ = 6.92 (d, 2H, *J* = 8.5 Hz), *δ*_C_ = 113.5 and *δ*_H_ = 8.12 (d, 2H, *J* = 8.5 Hz), *δ*_C_ = 130.5] with the ^2^*J*_H,C_ HMBC correlation among the proton at *δ*_H_ = 6.92 with the unprotonated carbon at *δ*_C_ = 164.1 bearing the OCH_3_ group and the ^3^*J*_H,C_ HMBC correlation among the proton at *δ*_H_ = 8.12 with the unprotonated carbon (CO) at *δ*_C_ = 186.6 (Figures S10–S14). However, the HMBC spectrum also showed a ^3^*J*_H,C_ correlation between a CH_3_ group [*δ*_H_ = 3.33 (s, 3H); *δ*_C_ = 32.0] linked to secondary nitrogen and the thiocarbonyl functional group at *δ*_C_ = 195.1, which allowed the combination of the *p*-methoxylbenzoyl portion with the *N*-methylthiocarbonyl unit, according to the proposed structure.

Polyaurine A (**5**, RT = 28.62 min) represents one of the major constituents of PAB2, as shown by the ^1^H NMR spectrum in CDCl_3_ of the analyzed fraction. This secondary metabolite was tentatively assigned by comparing the recorded spectroscopic means with those reported in our previous work [48]. Indeed, the HR-MS spectrum (Figure S5) associated with this peak showed a protonated molecular ion at *m*/*z* 266.1134 [M + H]^+^, which corresponds to the molecular formula C_12_H_15_N_3_O_4_. Instead, the HRMS^2^ profile showed the fragmentation pattern at *m*/*z* 135.0 (base peak) and *m*/*z* 234.0, all in good agreement with the reported information for polyaurine A [48]. Furthermore, the 2D NMR spectra (Figures S10–S14) showed long range ^1^H-^13^C correlations of the two aromatic methine groups [*δ*_H_ = 6.89 (d, 2H, *J* = 8.2 Hz), *δ*_C_ = 113.0, and *δ*_H_ = 8.17 (d, 2H, *J* = 8.2 Hz), *δ*_C_ = 131.1] with the quaternary carbons at *δ*_C_ = 130.7 and 162.4 belonging to the *p*-OCH_3_ benzene ring and a further correlation between the proton *δ*_H_ = 8.17 with the carbonyl at *δ*_C_ = 177.9, corroborating the presence of the *p*-methoxybenzoyl fragment (base peak at *m*/*z* 135.0). Interestingly, we observed two downfield protons at *δ*_H_ = 9.30 (1H, brs, NH) and *δ*_H_ = 10.59 (1H, brs, NH) as well as the additional N-CH_3_ (*δ*_H_ = 3.52, s, 3H; *δ*_C_ = 32.2) and -OCH_3_ (*δ*_H_ = 3.86, s, 3H; *δ*_C_ = 53.7) groups, for which HMBC correlations were, with two unprotonated sp^2^ carbons, in close agreement with both the guanidine and carbamate units of this metabolite.

Compound **6** was tentatively defined as polycarpathiamine B [49]. The chromatographic peak at RT = 29.47 min contained the [M + H]^+^ ion at *m*/*z* 236.0487, which is associated with the molecular formula C_10_H_9_N_3_SO_2_ (Figure S6). Despite the first structure elucidation of **6** being based on NMR spectra recorded in deuterated DMSO [49], in our previous work [48], the chemical shifts of polycarpathiamine B were recorded in CDCl_3_, and these are completely superimposable to those evidenced in PAB2 (Figures S10–S14).

This study also allowed the putative identification in the PAB2 fraction of polyaurine B (**8**, RT = 33.28) and its derivative **7** (RT = 30.87 min). As for compound **8**, the available literature [48] completely agrees with the proposed molecular formula C_12_H_13_N_3_SO_3_ proposed for the peak [M + H]^+^ at *m*/*z* 280.0750 (Figure S8). Likewise, the ^1^H NMR spectrum (Figure S9) of PAB2 clearly showed the diagnostic signals of the aromatic protons of **8** at *δ*_H_ = 6.96 (d, 2H, *J* = 7.9 Hz) and *δ*_H_ = 7.88 (d, 2H, *J* = 7.9 Hz). The latter showed a key HMBC correlation with the unprotonated carbon at *δ*_C_ = 187.1 belonging to the 1,2,4-thiadiazole ring of the marine metabolite (Figures S10–S14). Analogously, the chromatographic peak at RT = 30.87, which was associated with the ion peak [M + H]^+^ at *m*/*z* 222.0695 and the molecular formula C_10_H_12_N_3_SO, was identified as compound **7**, which represents a strictly correlated derivative of polyaurine B in which the carbamate functional group resulted as hydrolyzed (Figures S7, S10–S14). Compound **7** has already been reported since it is a crucial intermediate in the synthesis of polyaurine B [52].

### 2.4. Effect of PAB2 on LPS-Stimulated J774 Cells

**Figure 5:**
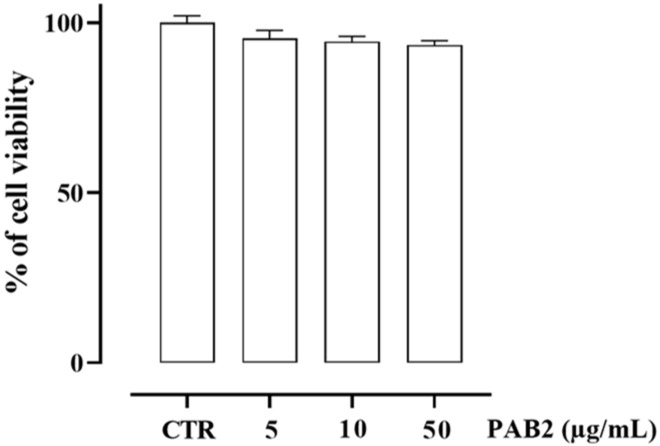
Effect of PAB2 on cell viability in J774 cells. Cell proliferation was measured using the MTT assay and evaluated at 24 h. Data are reported as % of cell viability and expressed as means ± SEM. Each experiment (*n* = 3) was run in quadruplicate.

**Figure 6:**
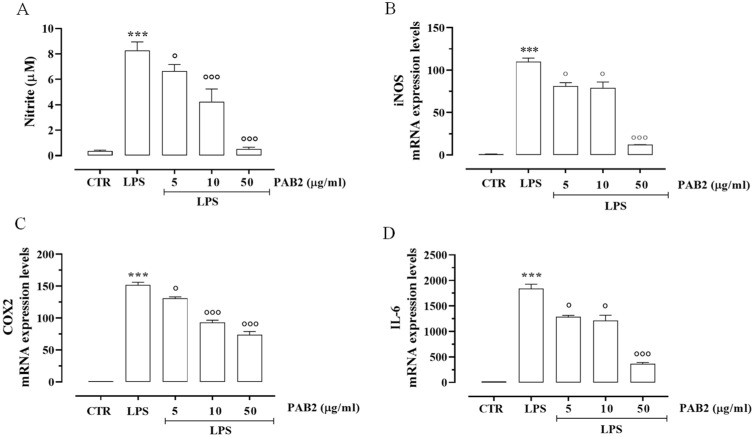
Anti-inflammatory effect of PAB2 in J774 cells. (**A**) Effect of different concentrations of PAB2 (5, 10, and 50 µg/mL) on the production of NO in J774 cells stimulated with LPS (1 µg/mL) and on the mRNA expression levels of (**B**) iNOS, (**C**) COX2, and (**D**) IL-6. Data are reported as µM or mRNA expression levels and expressed as means ± SEM. Each bar represents the mean ± SEM of *n* = 3 separate experiments run in triplicate. *** *p* < 0.001 vs. CTR, ° *p* < 0.05; °°° *p* < 0.001 vs. LPS.

First, we assessed the cytotoxicity of PAB2 in J774 cells. In order to address this issue, J774 cells were treated with PAB2 at concentrations of 5, 10, or 50 μg/mL. PAB2 was not cytotoxic at the concentrations tested (Figure 5). Therefore, the aforementioned concentrations were used to examine the in vitro anti-inflammatory activity of PAB2. More in detail, the effect of PAB2 was assessed on J774 cells stimulated with LPS, an in vitro method widely used to evaluate the anti-inflammatory activity. PAB2 significantly inhibited LPS-induced NO production in a concentration-dependent manner (Figure 6A). As NO represents a parameter of macrophage activation and inducible nitric oxide synthase (iNOS) induction, its expression was measured coupled to cyclooxygenase-2 (COX2) and interleukin-6 (IL6). LPS treatment promoted iNOS, COX2, and IL6 mRNA expression, which was markedly reduced following the treatment with PAB2 (Figure 6B–D).

### 2.5. PAB2 Induces Anti-Inflammatory Effect In Vivo

**Figure 7:**
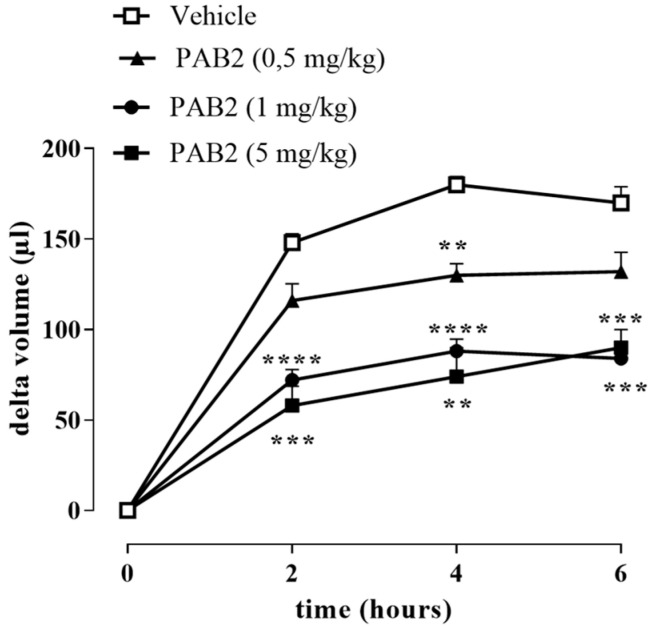
Effect of PAB2 on carrageenan-induced mouse paw edema. PAB2 (0.5, 1, and 5 mg/kg, i.p.) significantly reduces paw edema development. Data are reported as delta volume (µL) and expressed as means ± SEM (*n* = 5). ** *p* < 0.01, *** *p* < 0.001, and **** *p* < 0.0001 vs. vehicle.

**Figure 8:**
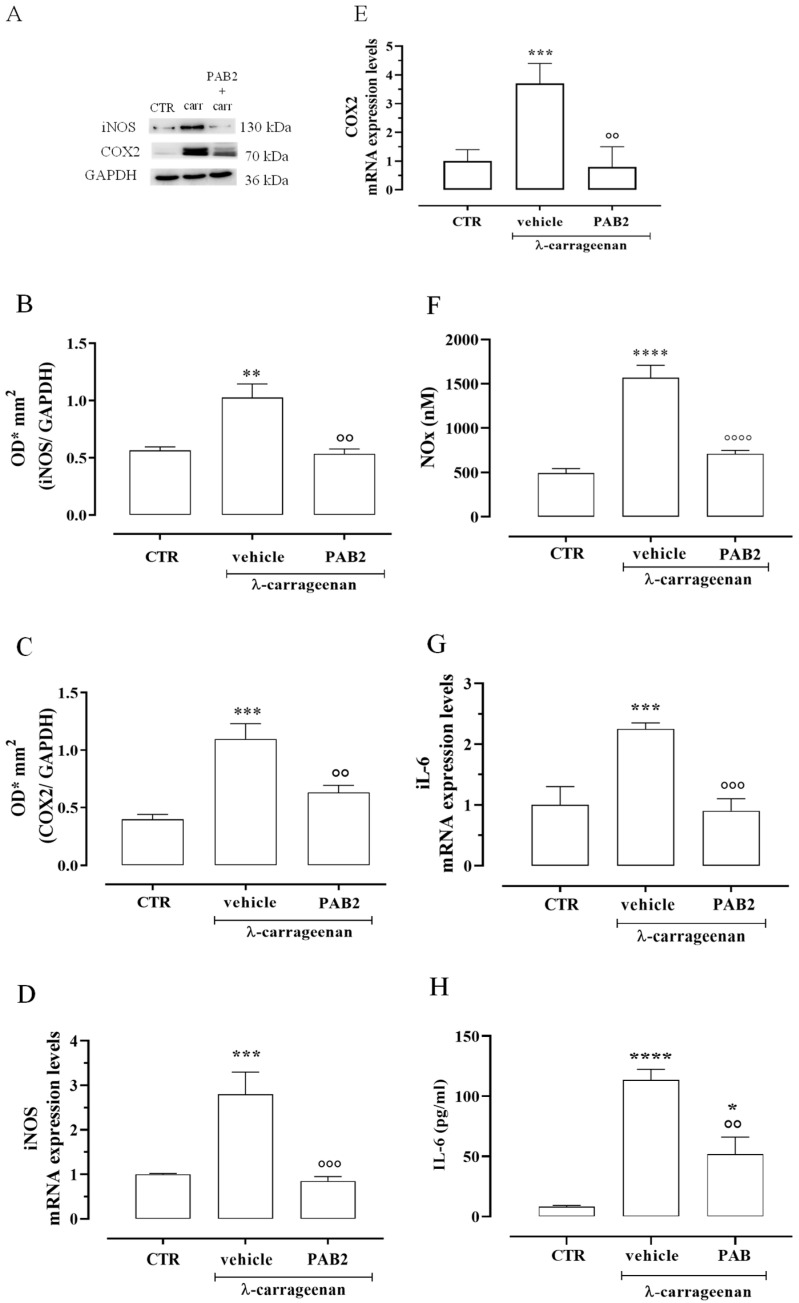
Anti-inflammatory effect of PAB2 in vivo model of carrageenan-induced paw edema. (**A**) Representative western blot for iNOS and COX2 in mouse hind paw. The protein expression of (**B**) iNOS and (**C**) COX2 is up-regulated after 6 h from λ-carrageenan injection compared to control (** *p* < 0.01 and *** *p* < 0.001); intraperitoneal administration of PAB2 (1 mg/kg) significantly reduces carrageenan-induced effect (°° *p* < 0.01 vs. λ-carrageenan). Data are reported as O.D.* mm^2^ and expressed as means ± SEM (*n* = 5). The mRNA expression of (**D**) iNOS and (**E**) COX2 is up-regulated after 6 h from λ-carrageenan injection compared to control (*** *p* < 0.001); intraperitoneal administration of PAB2 (1 mg/kg) significantly reduces carrageenan-induced effect (°° *p* < 0.01 and °°° *p* < 0.001 vs. carrageenan). Data are reported as mRNA expression levels and expressed as means ± SEM (*n* = 5). (**F**) NOx production is significantly increased after 6 h from λ-carrageenan injection compared to control (**** *p* < 0.0001); intraperitoneal administration of PAB2 (1 mg/kg) significantly reduces carrageenan-induced effect (°°°° *p* < 0.0001 vs. λ-carrageenan). Data are reported as nM and expressed as means ± SEM (*n* = 5). (**G**) IL-6 mRNA is up-regulated after 6 h from λ-carrageenan injection compared to control (*** *p* < 0.001); intraperitoneal administration of PAB2 (1 mg/kg) significantly reduces carrageenan-induced effect (°°° *p* < 0.01 vs. λ-carrageenan). Data are reported as mRNA expression levels and expressed as means ± SEM (*n* = 5). (**H**) IL-6 levels are up-regulated after 6 h from λ-carrageenan injection compared to control (* *p* < 0.05 and **** *p* < 0.0001); intraperitoneal administration of PAB2 (1 mg/kg) significantly reduces carrageenan-induced effect (°° *p* < 0.01 vs. λ-carrageenan). Data are reported as pg/mL and expressed as means ± SEM (*n* = 5).

The anti-inflammatory effect of PAB2 was evaluated in vivo in carrageenan-induced mouse paw edema for up to 6 h. Intraperitoneal administration of PAB2 (0.5, 1, and 5 mg/kg, i.p.) significantly reduced the edema formation (Figure 7; ** *p* < 0.01, *** *p* < 0.001, and **** *p* < 0.0001 vs. vehicle). More in detail, the doses of 1 mg/kg and 5 mg/kg significantly reduced edema to the same extent and at all time points (2, 4, and 6 h). Based on this latter result, the dose of 1 mg/kg was selected as optimal for the subsequent molecular studies. Of note, 6 h after the intraplantar injection of λ-carrageenan, the expression of both iNOS and COX2 in terms of mRNA and protein was markedly increased compared to control mice (Figure 8A–E; ** *p* < 0.01 and *** *p* < 0.001). Interestingly, PAB2 at the dose of 1 mg/kg significantly reduced iNOS and COX2 expression, recovering to the values of the control (Figure 8A–E; °° *p* < 0.01 and °°° *p* < 0.001). Next, we monitored the NOx amount in paw exudates as an index of vascular permeability [53]. Notably, PAB2 significantly reduced the increase in the NOx amount induced by λ-carrageenan (Figure 8F; **** *p* < 0.0001 vs. CTR and °°°° *p* < 0.0001 vs. λ-carrageenan).

In the same way, the increase in IL-6 mRNA observed 6 h after the intraplantar injection of λ-carrageenan was reverted by PAB2 (Figure 8G; *** *p* < 0.001 vs. CTR and °°° *p* < 0.001 vs. carrageenan). This data correlated well with the evaluation of IL-6 levels in mouse paw exudates. PAB2 significantly reduced the increase in the IL-6 amount observed in paw exudates 6 h after the intraplantar injection of λ-carrageenan (Figure 8H; * *p* < 0.05 and **** *p* < 0.0001 vs. CTR and °° *p* < 0.01 vs. λ-carrageenan).

## 3. Discussion

The marine environment is a very promising resource for the discovery of bioactive compounds that could have significant applications in drug development. Within this context, a comprehensive analysis was conducted on the components extracted from Indonesian *P. aurata*, leading to the identification of a novel donor of H_2_S. Among examined fractions of butanol extract of *P. aurata*, PAB2 was the best compared to the others in releasing H_2_S in solution, i.e., in a cell-free assay.

Eight main chemical constituents have been recognized in the PAB2 fraction. Their identity has been tentatively assigned through an extensive 2D NMR analysis, the full scan HRMS data, which provided the precursor ion mass, the HRMS^2^ fragmentations pattern, and by comparing these means with the available literature. The compounds were arranged based on their retention time (RT), as shown in Table 1 and Figure 3. Table 1 and Figure 3 indicate the tentatively identified compounds along with their retention times, experimental *m*/*z* in positive ionization mode, and MS/MS fragments. The most dereplicated compounds (**1**–**6**) show a *p*-methoxybenzoyl-related structure, which is also clearly responsible for the fragment peak *m*/*z* = 135.0 in their MS/MS spectra (Figures S1–S6). Moreover, in the downfield region (*δ*_H_ 6.5–8.6) of ^1^H NMR spectrum in CDCl_3_ (Figure S9) of PAB2, many aromatic signals as doublet resonances and the coupling constant patterns are observed, which are strictly related to the presence of *para*-substituted benzene ring. Moreover, spectrometric and spectroscopic analysis of PAB2 also allowed the putative identification of the heteroatom-rich metabolite polyaurine B (**8**) and its strictly correlated derivative, compound **7**, characterized by the loss of carbamate moiety. Our study is the first example of naturally occurring compound **7**, which is most likely obtained during the tunicate extraction and/or purification procedures due to the use of the aqueous medium.

Thus, the dereplication of PAB2 putatively provided at least four different sulfur-containing secondary metabolites (compounds **3** and **6**–**8**). Among the detected compounds, **3** is endowed with a thioamide function already described as an H_2_S-releasing chemical moiety [54] and also used for the design of new anti-inflammatory agents [16,55]. In this regard, ATB-346, an H_2_S-releasing naproxen, exerts a higher anti-inflammatory effect coupled with lower gastro toxicity compared with the parent drug [30].

The scientific literature largely reports inorganic salts such as sodium hydrosulfide (NaHS), sodium sulfide (Na_2_S), and calcium sulfide (CaS) to be exogenous sources of H_2_S despite several chemical limitations restricting their use in clinical settings (e.g., the air oxidation of hydrogen sulfide ions (HS^−^), which lowers the H_2_S concentration in the solution) [56]. Among the organic H_2_S donors, the most widely studied is a phosphinodithioate derivative named GYY4137 (morpholin-4-ium 4-methoxyphenylmorpholinophosphinodithioate), which decomposes spontaneously in an aqueous medium to release H_2_S over a long period [57]. However, other chemical scaffolds such as dithiolethiones and thioamides have been demonstrated to act as potent H_2_S-releasing moieties, such that they are largely used for synthesizing multifunctional drugs, even though the releasing mechanisms are not clear [54,58].

In the cell-free assay, PAB2 displayed the best profile as an H_2_S donor; indeed, the release in solution from PAB2 was remarkably elevated at the higher concentrations, reaching a value of 1.7 ± 0.017 µM of H_2_S at the highest concentration tested, hence we moved to the cell assay. First, we examined cell viability after treating J774 murine macrophages with PAB2. PAB2 was non-cytotoxic at concentrations of 5, 10, and 50 μg/mL, and these concentrations were used for subsequent experiments. Interestingly, the release of H_2_S detected following incubation of J774 murine macrophages with PAB2 was markedly higher and persisted for up to 2 h. Overall, these data indicate that PAB2 is an exogenous source of H_2_S.

It should be considered that H_2_S donors with different release rates might induce quantitative, as well as qualitative, variance in cellular responses [23,59]. Thus, the bell-shaped properties of H_2_S provide a useful framework to reconcile some of the controversies regarding H_2_S functions. However, the complexities of the temporal relationship between H_2_S donation and its effects remain to be further explored. Various classes of H_2_S donors have been tested in multiple models of inflammation. The results revealed that H_2_S exerts cytoprotective and anti-inflammatory effects, including the inhibition of multiple pro-inflammatory signaling pathways and a reduction in the production of reactive oxygen and nitrogen species [11,26,60].

Therefore, based on the functional studies defining PAB2 as an H_2_S donor, its potential anti-inflammatory effect was evaluated in in vitro and in vivo models of inflammation.

In order to address this issue, we used J774 murine macrophages. Indeed, macrophages play an essential role in immune responses and protect the body from various pathogens through phagocytosis [61]. LPS, outer cell membrane molecules of Gram-negative bacteria, are well-known endotoxins that can activate macrophages to generate many inflammatory factors [62]. Macrophages activated by LPS from *Escherichia coli* express pro-inflammatory enzymes, including iNOS and COX2. iNOS-derived NO plays a major role in inflammation [63,64]. IL-6 is a proinflammatory cytokine that plays a role in the maintenance of many homeostatic functions. However, its overexpression causes various inflammatory disorders [65]. For these reasons, we examined the effect of PAB2 on LPS-stimulated inflammatory enzymes and mediators. PAB2 significantly reduced the expression of iNOS and COX2 induced by LPS in J774 murine macrophages. In line, the production of NO and mRNA levels of IL-6 were significantly and markedly inhibited by PAB2 in J774 murine macrophages stimulated with LPS. Altogether, these findings demonstrated that PAB2 can slowly release H_2_S, modulating the pro-inflammatory action induced by LPS.

The proof of concept of the anti-inflammatory effect of PAB2 was given by using the classical model of acute inflammation, i.e., injecting λ-carrageenan in the hind paw of mice to generate edema. The anti-inflammatory effect of PAB2 was studied by using three different doses administered i.p. 30 min before λ-carrageenan injection. The intraplantar administration of λ-carrageenan significantly increased the paw volume compared to basal values, whereas treatment with PAB2 significantly reduced carrageenan-induced inflammation at all doses tested. Among the higher doses of PAB2 used, i.e., 1 and 5 mg/kg, no difference was found in the reduction of edema, so the dose of 1 mg/kg was selected for the subsequent molecular study. After 6 h from λ-carrageenan injection, the significant increase in iNOS and COX2 expression as both mRNA and protein was coupled to an increase in IL-6 mRNA and NO levels compared to the control group. Interestingly, PAB2 pre-treatment reduced the expression of iNOS, COX2, and IL-6 as well as the NO amount. These results suggest that PAB2 inhibits acute inflammatory responses in vivo through the release of H_2_S.

Therefore, for the first time, we have identified a marine-derived natural matrix as a source of H_2_S, opening a new scenario in this field.

## 4. Materials and Methods

### 4.1. General Experimental Procedures

Solvents (water, methanol, chloroform, ethyl acetate, butanol, formic acid) and deuterated chloroform used for the extraction and purification procedures as well as for NMR analysis were purchased from Merck Life Science S.R.L. (St. Louis, MO, USA) and were used without further purification. The ^1^H (700 MHz) and 2D NMR experiments (COSY, HSQC, HMBC) were carried out on a Bruker Avance Neo spectrometer (Bruker BioSpin Corporation, Billerica, MA, USA); chemical shifts were reported in parts per million (ppm) and referenced to the residual solvent signal (CDCl_3_: *δ*_H_ = 7.26; *δ*_C_ = 77.0). Homonuclear ^1^H connectivities were determined by COSY experiments, whereas ^1^H-^13^C long-range connectivities were determined by gradient 2D HMBC experiments optimized for a ^2,3^*J* of 8 Hz [66]. LC-HRMS analyses were carried out on an Ultimate 3000 quaternary LC system coupled to a hybrid linear ion trap LTQ Orbitrap XL™ FTMS equipped with an ESI source (Thermo Fisher, San Josè, CA, USA). Chromatographic analysis was performed on a Kinetex C18 column (2.6 μm, 100 Å, 4.6 × 100 mm; Phenomenex, Torrance, CA, USA) kept at room temperature.

### 4.2. Collection, Extraction, and Fractionation of the Ascidian P. aurata

Several specimens of the solitary ascidian *P. aurata* were collected along the coast of Indonesia (Siladen, 1°37′41″ N 124°48′01″ E) in autumn and immediately frozen after collection. The identification of each sample was performed by Dr. Masteria Yunovilsa Putra; a voucher of *P. aurata* was deposited at the Department of Pharmacy, University of Naples Federico II, Napoli, Italy. The animals were thawed, homogenized, and exhaustively extracted three times with methanol (3 × 400 mL) and then twice with chloroform (2 × 400 mL). The different extracts were combined and concentrated under reduced pressure; the resulting organic was dissolved in water and partitioned with butanol. The butanol soluble material was chromatographed by medium-pressure liquid chromatography (MPLC) over reversed-phase silica gel using an increasing gradient elution: H_2_O/MeOH 9:1 → H_2_O/MeOH 7:3 → H_2_O/MeOH 1:1 → H_2_O/MeOH 3:7 → H_2_O/MeOH 2:8 → MeOH 100%. The eluted fractions were monitored by preliminary 1H NMR, and five fractions were obtained (PAB1–PAB5). The most active fraction, PAB2, was further analyzed by LC-HRMS and by mono- and bidimensional NMR spectroscopy.

### 4.3. LC-HRMS/MS Profiling of P. aurata

LC-HRMS experiments were carried out on the Dionex Ultimate 3000 system, which included a solvent reservoir, in-line degasser, quaternary pump, and refrigerated autosampler and column oven, coupled to a hybrid linear ion trap LTQ Orbitrap XL^TM^ Fourier Transform MS (FTMS) equipped with an ESI ION MAX^TM^ source (Thermo Fisher, San Josè, CA, USA). Chromatographic separation was performed on a Kinetex C18 column (2.6 μm, 100 Å, 4.6 × 100 mm; Phenomenex, Torrance, CA, USA) kept at room temperature and eluted at 0.5 mL/min with water (eluent A) and methanol (eluent B), both containing 0.1% formic acid. A gradient elution was used: 5% B hold for the first 3 min, 5–95% B over 32 min, and hold for 5 min. The volume injected was set at 5 μL.

HR full MS experiments (positive ions) were carried out in the mass ranges *m*/*z* 100–1000 at a resolving power of 30,000, with a spray voltage of 4.8 kV, a capillary temperature of 275 °C, a capillary voltage of 13 V, a sheath gas, and an auxiliary gas flow of 51 and 1 (arbitrary units), respectively, and a tube lens voltage of 80 V [67].

Data were recorded with a data-dependent acquisition mode, in which the most intense ions in the full-scan mass spectrum were subjected to high-resolution tandem mass spectrometry (HRMS/MS) analysis. HRMS/MS scans were achieved for the selected ions with collision-induced dissociation (CID), an isolation width of 3.00 Da, a normalized collision energy of 25 units, an activation Q of 0.250 units, and an activation time of 30 ms.

Elemental formulae of ions contained in HRMS and HRMS^2^ spectra were assigned by using the mono-isotopic ion peak of each ion cluster and the Xcalibur 2.2 software, setting a mass tolerance of 5 ppm.

### 4.4. Hydrogen Sulfide Measurement in a Cell-Free Assay

The ability of all five PAB1, PAB2, PAB3, PAB4, and PAB5 fractions to release H_2_S was evaluated by a fluorescence-based assay using the fluorescent probe sulfidefluor-7-acetoxymethyl ester (SF7-AM; Sigma, Milan, Italy) [68]. The SF7-AM was dissolved in dimethylsulfoxide (DMSO) and used at a final concentration of 10 μM. The samples were prepared in the concentration range of 1–500 µg/mL; more in detail, the extracts were dissolved in DMSO at the starting concentration of 10 mg/mL and then diluted in pure distilled water at subsequent concentrations. SF7-AM was added to the samples, and the mixture was incubated in a black 96-well plate in the dark at 37 °C. The fluorescence was measured after 15 min of incubation with PAB1, PAB2, PAB 3, PAB4, and PAB5 (ex: 475 nm; em: 500–550 nm). PAB2 was the most effective for releasing H_2_S and thus was selected for the study. The release of H_2_S from PAB2 was monitored for up to 45 min and compared to the vehicle. The concentration of H_2_S released was determined through a calibration curve of Na_2_S (50 nM–200 μM). Results were expressed as means ± SEM (*n* = 3) and reported as µM.

### 4.5. Cell Culture

The murine monocyte/macrophage cell line J774 was from ATCC. J774 cells were grown in Dulbecco’s modified Eagle’s medium (DMEM; Biowhittaker) and cultured at 37 °C in humidified 5% CO_2_/95% air. The cells were plated in 24-well culture plates (Falcon, SIAL, Rome, Italy) at a density of 2.5 × 10^6^ cells/mL/well and allowed to adhere for 2 h. Thereafter, the medium was replaced with fresh medium, and cells were activated by lipopolysaccharide (LPS 1 µg/mL) from *E. coli* (Sigma, Milan, Italy) for 18 h in the presence or absence of different concentrations of test compounds. The culture medium was then removed and centrifuged, and the supernatant was used for the determination of nitrite (NO^2−^) production.

Cell viability (>95%) was determined with the MTT assay [69].

### 4.6. Hydrogen Sulfide Measurement in J774 Cells

J774 cells were incubated in DMEM without phenol red for 30 min at 37 °C. After incubation, the medium was replaced with a medium containing SF7AM (10 μM) for 30 min at 37 °C. Thereafter, cells were treated with different concentrations of PAB2 (30, 100, and 300 μg/mL) to measure live H_2_S production. The fluorescence was measured every minute from basal (0) to 5 min and at 15-min intervals up to 2 h. Results were expressed as means ± SEM (*n* = 3) and reported as fluorescence intensity.

### 4.7. Measurement of NO Production in J774 Cells

NO^2−^ levels in culture media from J774 macrophages were measured 18 h after LPS with the Griess reaction, as previously described [70,71]. Results are expressed as µM of NO^2−^ and represent the means ± SEM of *n* = 3 experiments run in triplicates.

### 4.8. RNA Purification and Quantitative Real-Time PCR

Total RNA was extracted from cells or mouse paw homogenates using the QIAzol Lysis Reagent according to the manufacturer’s instructions (Qiagen, Hilden, Germany). Spectrophotometric quantization of each purified RNA was estimated using a Nanodrop apparatus (Thermo Fisher Scientific, Waltham, MA, USA). The purified RNA was considered DNA and protein-free if the ratio between readings at 260/280 nm was ≥1.8. Isolated mRNA was reverse-transcribed using iScript Reverse Transcription Supermix for RT-qPCR (Bio-Rad, Segrate, Italy). qPCR was carried out in a CFX96 real-time PCR detection system (Bio-Rad) with the use of SYBR Green Master Mix kit (Bio-Rad, Segrate, Italy) and the following primers: COX2 FW: GCGACATACTCAAGCAGGAGCA REV: AGTGGTAACCGCTCAGGTGTTG; iNOS FW: GAGACAGGGAAGTCTGAAGCAC REV: CCAGCAGTAGTTGCTCCTCTTC; and IL6 FW: TACCACTTCACAAGTCGGAGGC REV: CTGCAAGTGCATCATCGTTGTTC. The real-time PCR cycling protocol was (i) polymerase activation and DNA denaturation at 95 °C for 30 s; (ii) amplification for 40 cycles; (iii) denaturation for 15 s at 95 °C; (iv) annealing and extension for 30 s 60 °C; (v) plate read at 60 °C; and (vi) melt-curve analysis performed at 65–95 °C in 0.5 °C increments at 5 s/step. The housekeeping gene β-actin (FW: CATTGCTGACAGGATGCAGAAGG REV: TGCTGGAAGGTGGACAGTGAGG) was used as an internal control to normalize the CT values, using the 2^−ΔΔCt^ formula.

### 4.9. Animals

Animal care and experimental procedures in this study followed specific guidelines of the Italian and European Council law for experiments involving animals. All procedures were approved by the local animal care office (Centro Servizi Veterinari, University of Naples, Federico II) and carried out following ARRIVE guidelines [72,73] and EU recommendations (Directive 2010/63/EU) for experimental design and analysis in pharmacology care. The procedure was authorized by the Italian Health Ministry (code number: 950/2020-PR).

Male CD1 mice (35–38 g, Charles River, Calco, Italy) were housed in the animal care facility at the Department of Molecular Medicine and Medical Biotechnology, School of Medicine and Surgery, University of Naples, Italy. Animals were kept in a controlled environment with temperature (21 ± 2 °C), humidity (60 ± 10%), and 12 h light/dark cycles with free access to standard rodent chow and water.

### 4.10. Measurement of Mouse Paw Edema

PAB2 (0.5, 1, and 5 mg/kg) or vehicle was intraperitoneally administered in mice 30 min before the intraplantar injection of λ-carrageenan (0.1%). Edema formation was measured at scheduled time points, i.e., 0, 2, 4, and 6 h after the intraplantar injection by using a plethysmometer (Ugo Basile, Comerio, Italy). Data were calculated as means ± SEM (*n* = 5), and the edema formation was reported as an increase in paw volume (µL). In parallel, the same protocol was performed to obtain samples for molecular studies.

In detail, 6 h after the intraplantar injection of λ-carrageenan or vehicle (CTR), animals were euthanized, and paws were collected and frozen at −80 °C until assayed for RT-qPCR or western blot analysis. In another set of experiments, paws were harvested and centrifuged at 4000 rpm for 30 min; the exudates were collected with 100 µL of saline and used for NOx and IL-6 measurement.

### 4.11. Western Blotting Analysis

Paws were collected 6 h after the intraplantar injection of λ-carrageenan or vehicle (CTR), as previously described. Mouse paws were homogenized in modified RIPA buffer (50-mM Tris-HCl pH 8.0, 150-mM NaCl, 0.5% sodium deoxycholate, 0.1% sodium dodecyl sulfate, 1 mM EDTA, 1% Igepal) containing a protease inhibitors cocktail. Protein concentration was quantified by using Bradford assay using BSA as the standard (Bio-Rad Laboratories, Milan, Italy) [74]. Denatured proteins (70 μg) were separated on 8% sodium dodecyl sulfate-polyacrylamide gels and transferred to a polyvinylidene fluoride membrane. The membranes were blocked by incubation in phosphate-buffered saline (PBS) containing 0.1% *v*/*v* Tween 20 and 3% nonfat dried milk (AppliChem, Milan, Italy) for 1 h at room temperature and then incubated overnight at 4 °C with rabbit polyclonal anti-inducible nitric oxide synthase (iNOS; 1:1000; Novus, St. Louis, MO, USA) or mouse monoclonal anti-cyclooxygenase-2 (COX2; BD Transduction Laboratories, Franklin Lakes, NJ, USA). The membranes were extensively washed in PBS containing 0.1% *v*/*v* Tween-20 and then incubated with horseradish peroxidase-conjugated secondary antibody for 2 h at room temperature. Glyceraldehyde-3-phosphate dehydrogenase (GAPDH, 1:5000; Sigma Aldrich, Milan, Italy) was used as a housekeeping protein to normalize protein expression. Following incubation, membranes were washed and developed using ImageQuant-400 (GE Healthcare, Chicago, IL, USA). Data were expressed as optical density (OD)* mm^2^ and calculated as means ± SEM (*n* = 5 mice).

### 4.12. NOx Measurement in the Mouse Paw

Paw exudates were collected 6 h after the intraplantar injection of λ-carrageenan or vehicle (CTR), as previously described. Samples were incubated in a microplate with cadmium (Sigma-Aldrich; 50 mg per well) for 1 h to convert the inorganic anion nitrate (NO^3−^) to nitrite (NO^2−^). After centrifugation at 8000× *g*, total NOx content was determined using a fluorimetric method by a Promega GloMax Explorer (Madison, WI, USA) and calculated against a standard curve of sodium nitrite (NaNO_2_, 50–2000 nM; Sigma-Aldrich) [75,76]. Each independent experiment was performed in duplicate. Data were calculated as means ± SEM (*n* = 5 mice) and reported as nM.

### 4.13. IL-6 Measurement in the Mouse Paw

Paw exudates were collected 6 h after the intraplantar injection of λ-carrageenan or vehicle (CTR), as previously described. Samples were processed for the measurement of IL-6 levels as described in the manufacturer’s protocol ELISA Kit (Abcam, Cambridge, UK). Each independent experiment was performed in duplicate. Data were calculated as means ± SEM (*n* = 5 mice) and reported as pg/mL.

### 4.14. Statistical Analysis

The results were calculated as means ± SEM. Statistical analysis was performed using one-way ANOVA or two-way ANOVA followed by Bonferroni’s test as needed. *p* values less than 0.05 were considered significant.

## 5. Conclusions

Collectively, H_2_S donors would be therapeutically useful and expected to be anti-inflammatory in conditions of uncontrolled inflammation (typically, in the late stages of inflammatory disease). Even though this mediator has been widely investigated in the inflammatory field, recent efforts in the search for new chemical structures to control H_2_S release are paving the way, exploiting this pleiotropic gasotransmitter in other therapeutic fields.

In this view, marine extracts may represent a powerful tool since they contain an array of compounds, at varying abundance, providing a wide chemical space to be investigated in the search for active chemical scaffolds. Despite this, the isolation of pure metabolites, responsible for a certain biological effect, is a compelling challenge. However, several studies have shown that the overall activity of extracts as well as derived fractions can be higher than the summative activities of each compound due to synergistic, additive, or antagonistic effects [39,77]. For this reason, it is not rare to observe the loss of activity after the efforts spent for the isolation of individual constituents.

Many marine bioactive compounds have been shown to possess peculiar biological properties, but only a few have been tested in combination [77]. Here, for the first time, the potential of marine extract as a source of H_2_S has been demonstrated, highlighting its anti-inflammatory activity. Thus, our study reinforces the need to test fractions of multiple marine natural products, providing incredible opportunities to develop novel combinations with greater efficacy for the prevention and treatment of various diseases.

## Figures and Tables

**Table 1:** Assignment of parent and related fragment ions contained in HR full mass scan and HR CID MS^2^ spectrum of PAB2. Elemental formulae of the mono-isotopic ion peaks (*m*/*z*) are reported with double bond/ring equivalents (RDB) and errors (Δ, ppm).

Exp. MS (*m*/*z*)	Δ ppm, RDB	Formula	RT	Putative Compound 1
208.1078	−1.217, 5.5	C_10_H_14_N_3_O_2_^+^	15.53	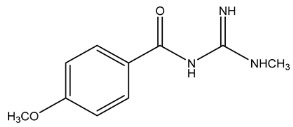
**Fragment ions**				
**Exp. MS (*m*/*z*)**	**Δ ppm, RDB**	**Formula**		**Structural hypothesis**
191.0816	0.502, 6.5	C_10_H_11_N_2_O_2_^+^		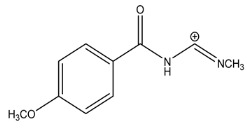
152.0705	−0.691, 4.5	C_8_H_10_NO_2_^+^		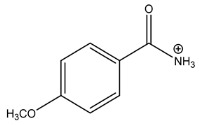
135.0439	−1.155, 5.5	C_8_H_7_O_2_^+^		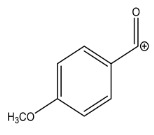
**Exp. MS (*m*/*z*)**	**Δ ppm, RDB**	**Formula**	**RT**	**Putative** **Compound 2**
194.0809	−1.390, 5.5	C_10_H_12_NO_3_^+^	20.91	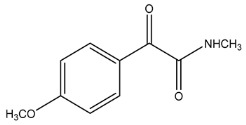
**Fragment ions**				
**Exp. MS (*m*/*z*)**	**Δ ppm, RDB**	**Formula**		**Structural hypothesis**
135.0439	−1.155, 5.5	C_8_H_7_O_2_^+^		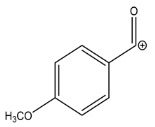
**Exp. MS (*m*/*z*)**	**Δ ppm, RDB**	**Formula**	**RT**	**Putative** **Compound 3**
210.0584	0.353, 5.5	C_10_H_12_NSO_2_^+^	24.79	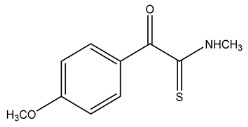
**Fragment ions**				
**Exp. MS (*m*/*z*)**	**Δ ppm, RDB**	**Formula**		**Structural hypothesis**
135.0439	−0.451, 5.5	C_8_H_7_O_2_^+^		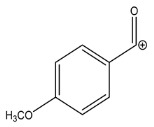
109.0648	0.078, 3.5	C_7_H_9_O^+^		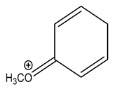
**Exp. MS (*m*/*z*)**	**Δ ppm, RDB**	**Formula**	**RT**	**Putative** **Compound 4**
195.0650	−1.155, 5.5	C_10_H_11_O_4_^+^	25.46	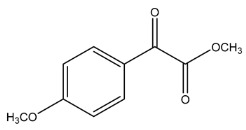
**Fragment ions**				
**Exp. MS (*m*/*z*)**	**Δ ppm, RDB**	**Formula**		**Structural hypothesis**
135.0440	−0.711, 5.5	C_8_H_7_O_2_^+^		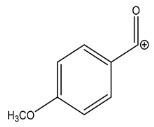
121.0283	−1.289, 5.5	C_7_H_5_O_2_^+^		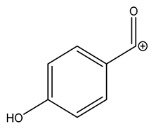
**Exp. MS (*m*/*z*)**	**Δ ppm, RDB**	**Formula**	**RT**	**Putative** **Compound 5**
266.1134	−0.498, 6.5	C_12_H_16_N_3_O_4_^+^	28.62	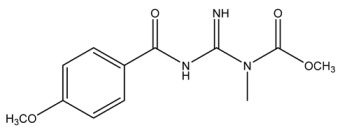
**Fragment ions**				
**Exp. MS (*m*/*z*)**	**Δ ppm, RDB**	**Formula**		**Structural hypothesis**
234.0869	−1.614, 7.5	C_11_H_12_N_3_O_3_^+^		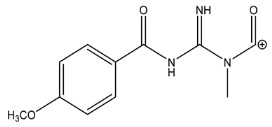
135.0437	−2.710, 5.5	C_8_H_7_O_2_^+^		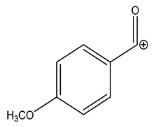
**Exp. MS (*m*/*z*)**	**Δ ppm, RDB**	**Formula**	**RT**	**Putative** **Compound 6**
236.0487	−0.567, 7.5	C_10_H_10_N_3_SO_2_^+^	29.47	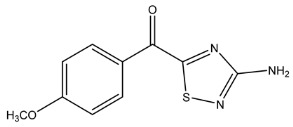
**Fragment ions**				
**Exp. MS (*m*/*z*)**	**Δ ppm, RDB**	**Formula**		**Structural hypothesis**
135.0439	−1.377, 5.5	C_8_H_7_O_2_^+^		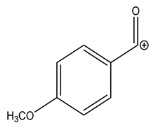
**Exp. MS (*m*/*z*)**	**Δ ppm, RDB**	**Formula**	**RT**	**Putative** **Compound 7**
222.0695	−0.447, 6.5	C_10_H_13_N_3_SO^+^	30.87	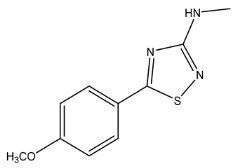
**Exp. MS (*m*/*z*)**	**Δ ppm, RDB**	**Formula**	**RT**	**Putative** **Compound 8**
280.0750	−0.102, 7.5	C_12_H_14_N_3_SO_3_^+^	33.28	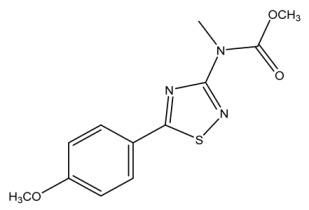
**Fragment ions**				
**Exp. MS (*m*/*z*)**	**Δ ppm, RDB**	**Formula**		**Structural hypothesis**
248.04918	1.436, 8.5	C_11_H_10_N_3_SO_2_^+^		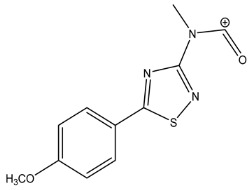

## Data Availability

Data will be made available on request.

## Supplementary Materials

The following supporting information can be downloaded at https://www.mdpi.com/article/10.3390/md21120641/s1: Figures S1–S8. LC-HRMS of compounds **1**–**8**: (A) Extracted ion chromatogram (XIC) at selected ions, (B) relevant HRMS, and (C) relevant HRMS^2^ spectra; Figure S9. ^1^H NMR spectrum (700 MHz) in CDCl_3_ of PAB2; Figure S10. COSY spectrum in CDCl_3_ of PAB2; Figure S11. Downfield region enlargement of COSY spectrum in CDCl_3_ of PAB2; Figure S12. HSQC spectrum in CDCl_3_ of PAB2; Figure S13. HMBC spectrum in CDCl_3_ of PAB2; Figure S14. Downfield region enlargement of HMBC spectrum in CDCl_3_ of PAB2.
