# Supplementary material for: Searching for Novel Sources of Hydrogen Sulfide Donors: Chemical Profiling of Polycarpa aurata Extract and Evaluation of the Anti-Inflammatory Effects
Source: Mar Drugs. 2023 Dec 15;21(12):641. doi: 10.3390/md21120641 (PMC10744941; doi:10.3390/md21120641)
Supplement: Supplementary file 1 [file marinedrugs-21-00641-s001.zip › marinedrugs-2695476-supplementary.pdf]

Searching for Novel Sources of Hydrogen Sulfide Donors: Chemical Profiling of *Polycarpa aurata* Extract and Evaluation of the Anti-inflammatory Effects

Marcello Casertano[1,†], Erika Esposito[1, †], Ivana Bello[1], Chiara Indolfi[2], Masteria Yunovilsa Putra[3], Lorenzo Di Cesare Mannelli[4], Carla Ghelardini[4], Marialuisa Menna[1], Raffaella Sorrentino[1], Giuseppe Cirino[1], Roberta d'Emmanuele di Villa Bianca[1]*, Concetta Imperatore[1]*, Elisabetta Panza[1,†], Emma Mitidieri[1,†]

1 Department of Pharmacy, School of Medicine and Surgery, University of Naples Federico II, Via D. Montesano 49, 80131 Naples, Italy; marcello.casertano@unina.it (M.C.); erika8esposito@gmail.com (E.E.); iv.bello.ib@gmail.com (I.B.); mlmenna@unina.it (M.M.); rafsorre@unina.it (R.S.); cirino@unina.it (G.C.); e.panza@unina.it (E.P.); emma.mitidieri@unina.it (E.M.)

2 Department of Molecular Medicine and Medical Biotechnology, School of Medicine and Surgery, University of Naples Federico II, Naples, Italy; chiara.indolfi@unina.it (C.I.)

3 Research Center for Vaccine and Drugs, Research Organisation for Healths, National Research and Innovation Agency (BRIN), Jalan Raya Jakarta-Bogor KM. 46, Cibinong 16911, Indonesia; masteria.yunovilsa.putra@brin.go.id (M.Y.P.)

4 Department of Neuroscience, Psychology, Drug Research and Child Health-NEUROFARBA-Pharmacology and Toxicology Section, University of Florence; lorenzo.mannelli@unifi.it (L.D.C.M); carla.ghelardini@unifi.it (C.G.)

Table of content

Figure S1. LC-HRMS of **1**: (**A**) Extracted ion Chromatogram (XIC) at the selected ion, (**B**) relevant HRMS and (**C**) relevant HRMS^2 spectra 2

Figure S2. LC-HRMS of **2**: (**A**) Extracted ion Chromatogram (XIC) at the selected ion, (**B**) relevant HRMS and (**C**) relevant HRMS^2 spectra 4

Figure S3. LC-HRMS of **3**: (**A**) Extracted ion Chromatogram (XIC) at the selected ion, (**B**) relevant HRMS and (**C**) relevant HRMS^2 spectra 5

Figure S4. LC-HRMS of **4**: (**A**) Extracted ion Chromatogram (XIC) at the selected ion, (**B**) relevant HRMS and (**C**) relevant HRMS^2 spectra 7

Figure S5. LC-HRMS of **5**: (**A**) Extracted ion Chromatogram (XIC) at the selected ion, (**B**) relevant HRMS and (**C**) relevant HRMS^2 spectra 8

Figure S6. LC-HRMS of **6**: (**A**) Extracted ion Chromatogram (XIC) at the selected ion, (**B**) relevant HRMS and (**C**) relevant HRMS^2 spectra 11

Figure S7. LC-HRMS of **7**: (**A**) Extracted ion Chromatogram (XIC) at the selected ion, (**B**) relevant HRMS and (**C**) relevant HRMS^2 spectra 13

Figure S8. LC-HRMS of **8**: (**A**) Extracted ion Chromatogram (XIC) at the selected ion, (**B**) relevant HRMS and (**C**) relevant HRMS^2 spectra 14

Figure S9. ^{1}H NMR spectrum (700 MHz) in $CDCl_3$ of PAB2 16

Figure S10. COSY spectrum in $CDCl_3$ of PAB2 16

Figure S11. Downfield region enlargement of COSY spectrum in $CDCl_3$ of PAB2 17

Figure S12. HSQC spectrum in $CDCl_3$ of PAB2; 17

Figure S13. HMBC spectrum in $CDCl_3$ of PAB2 18

Figure S14. Downfield region enlargement of HMBC spectrum in $CDCl_3$ of PAB2 18

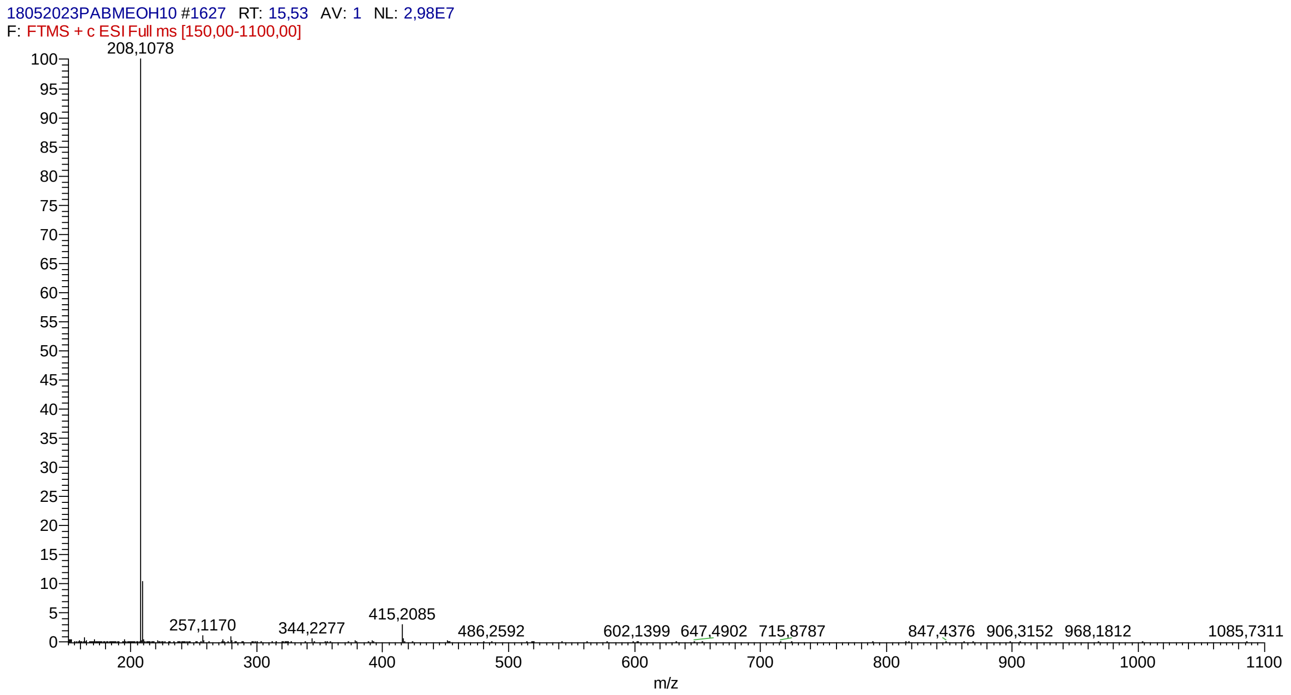
18052023PABMEOH10 #1627 RT: 15,53 AV: 1 NL: 2,98E7
F: FTMS + c ESI Full ms [150,00-1100,00]
208,1078
257,1170
344,2277
415,2085
486,2592
602,1399
647,4902
715,8787
847,4376
906,3152
968,1812
1085,7311
m/z

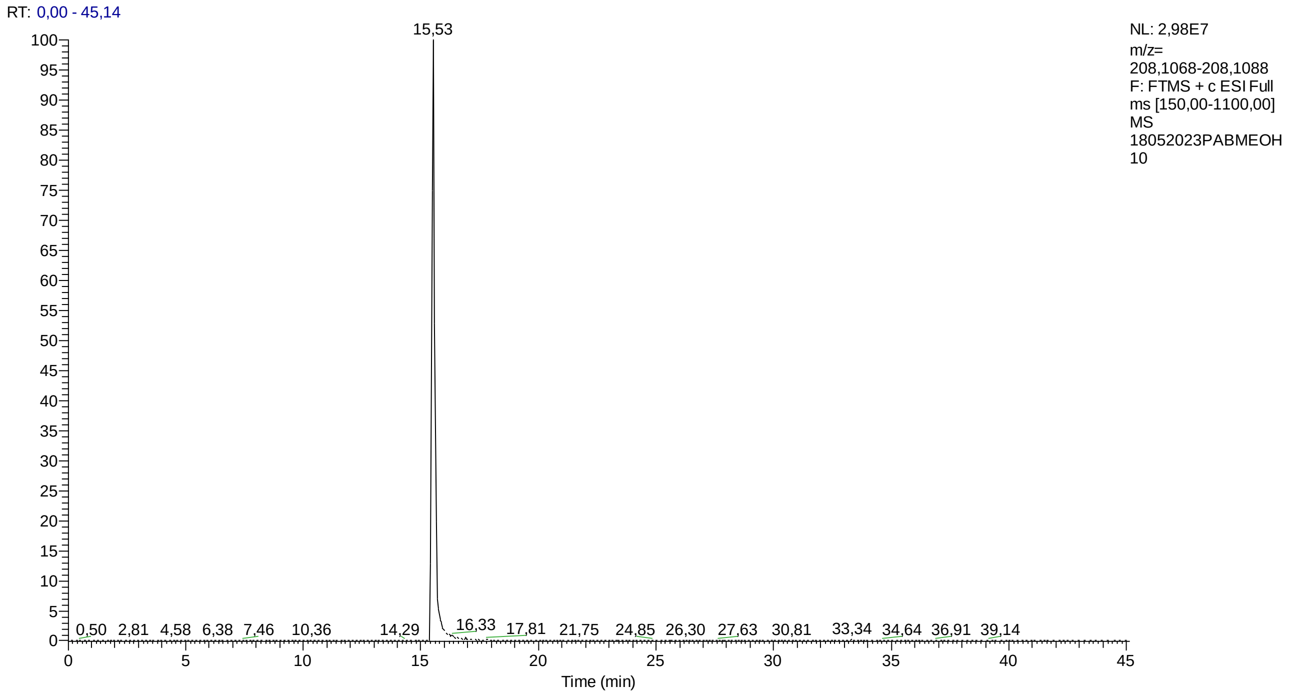
RT: 0,00 - 45,14
15,53
NL: 2,98E7
m/z=
208,1068-208,1088
F: FTMS + c ESI Full
ms [150,00-1100,00]
MS
18052023PABMEOH
10
0,50 2,81 4,58 6,38 7,46 10,36 14,29 16,33 17,81 21,75 24,85 26,30 27,63 30,81 33,34 34,64 36,91 39,14
Time (min)

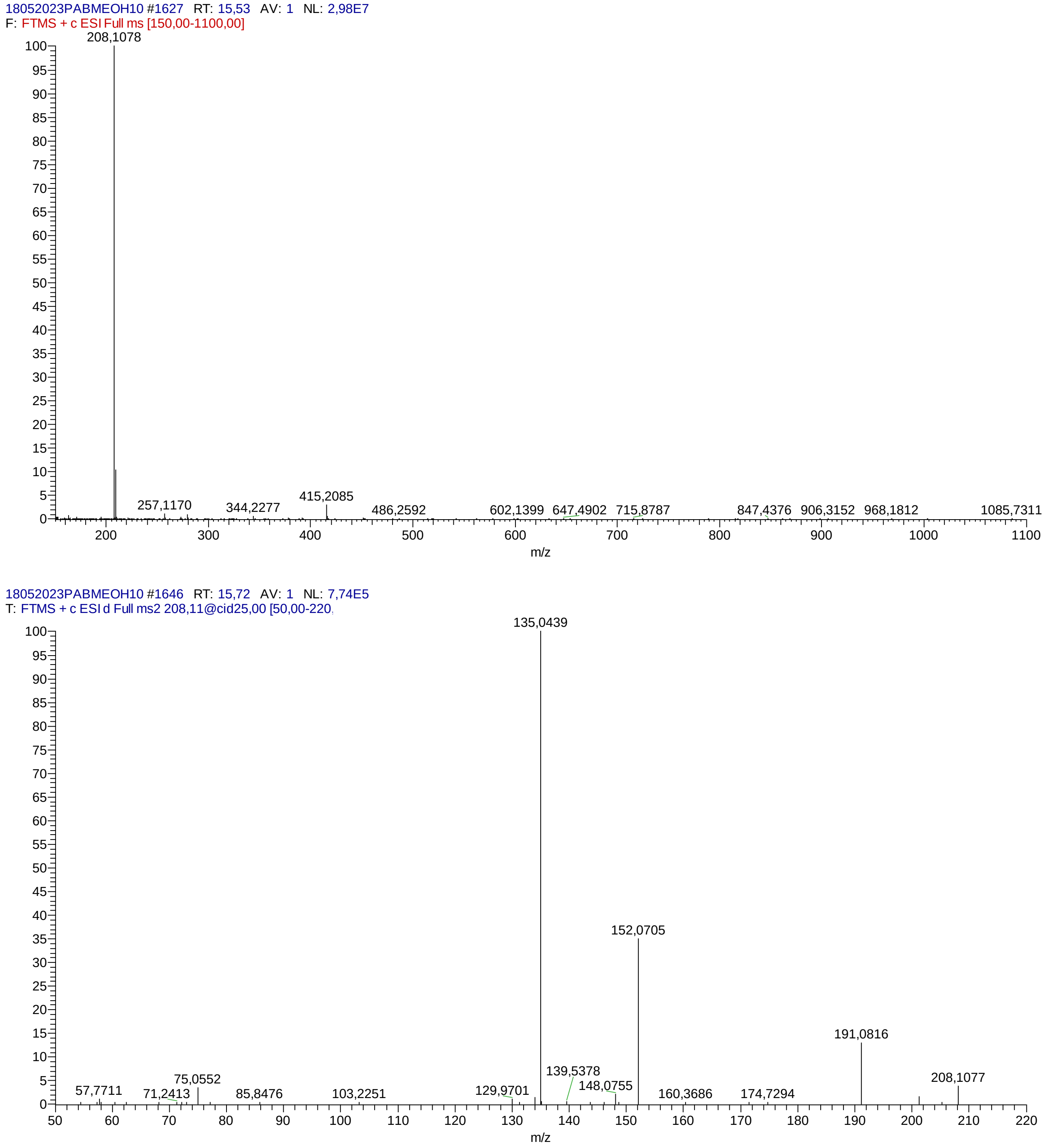

Figure S1. LC-HRMS analyses of **1** on an LTQ Orbitrap XL™ Hybrid FT Mass Spectrometer system: (**A**) Extracted ion Chromatogram (XIC) obtained by selecting the ion at *m/z* 208.1078, (**B**) relevant HRMS spectrum and (**C**) relevant HRMS^2 spectrum (Collision Induced Dissociation mode) obtained by using the ion at *m/z* 208.1 as precursor.

RT: 0,00 - 45,14

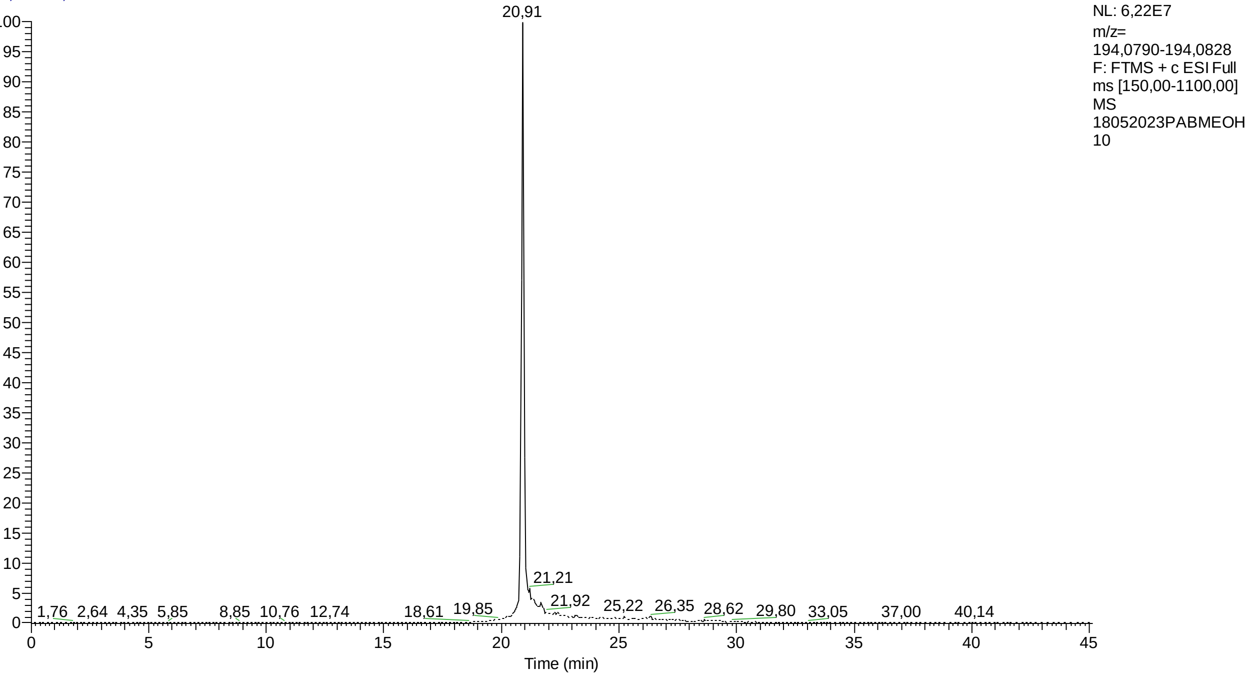

18052023PABMEOH10 #2185 RT: 20,84 AV: 1 NL: 3,56E7
F: FTMS + c ESI Full ms [150,00-1100,00]

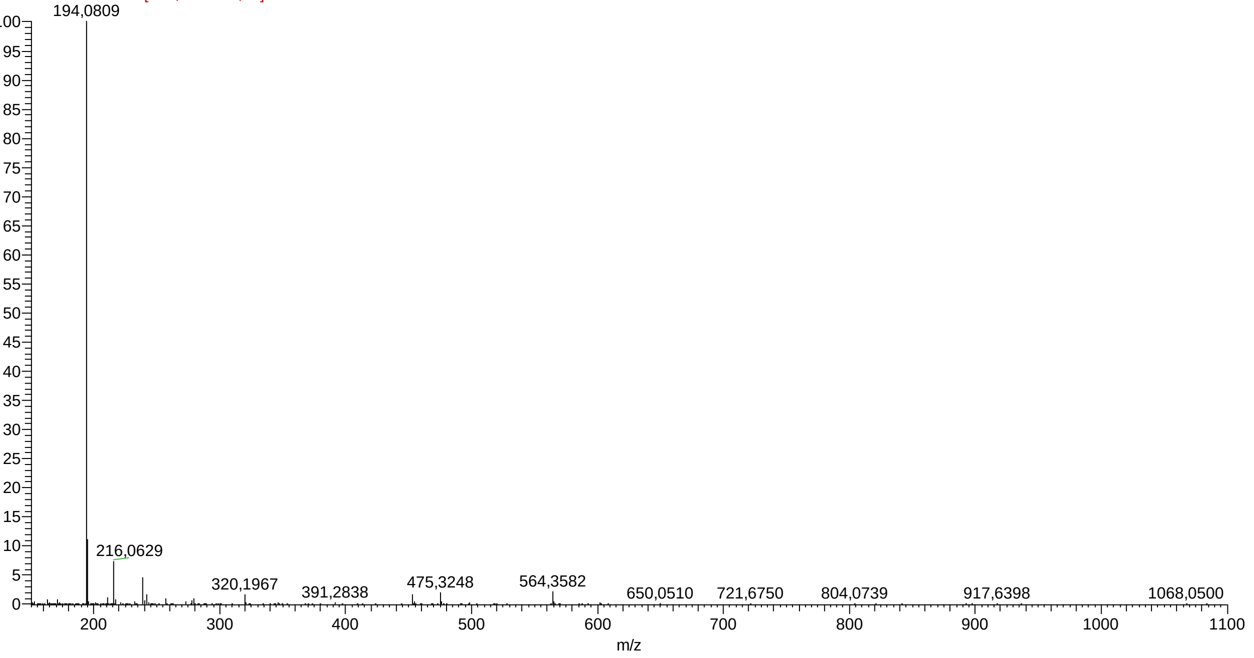

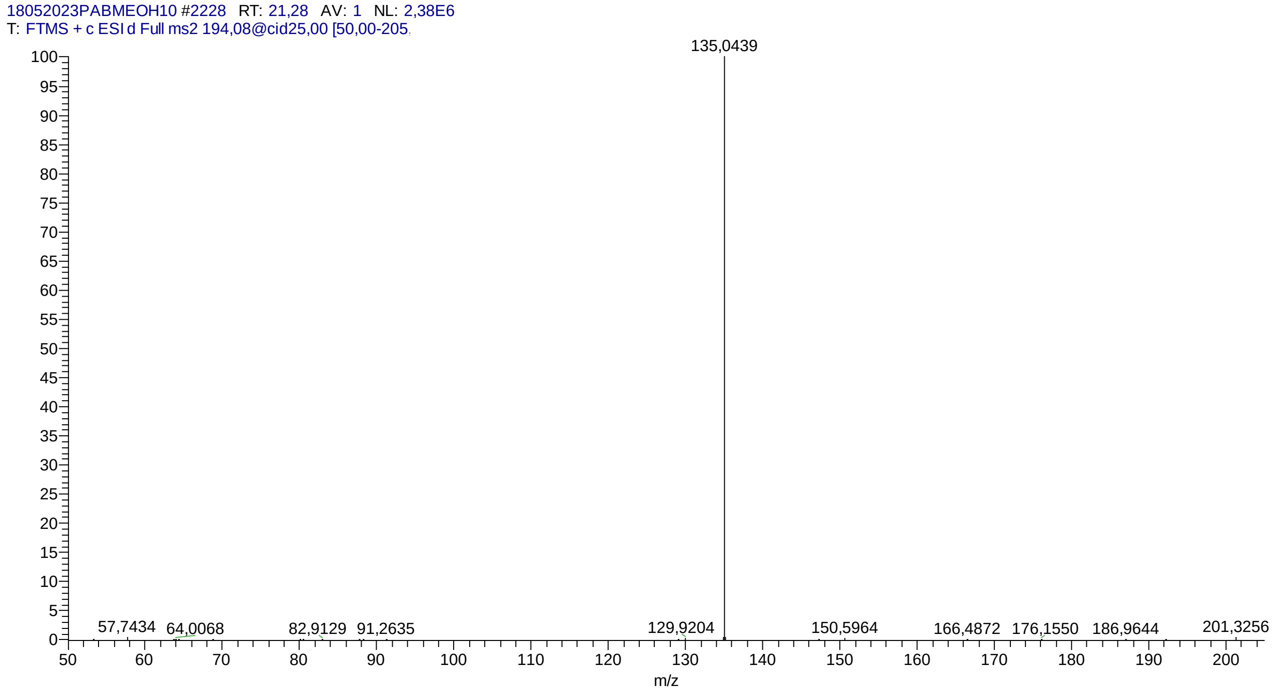

Figure S2. LC-HRMS analyses of **2** on an LTQ Orbitrap XL™ Hybrid FT Mass Spectrometer system: (**A**) Extracted ion Chromatogram (XIC) obtained by selecting the ion at *m/z* 194.0809, (**B**) relevant HRMS spectrum and (**C**) relevant HRMS² spectrum (Collision Induced Dissociation mode) obtained by using the ion at *m/z* 194.1 as precursor.

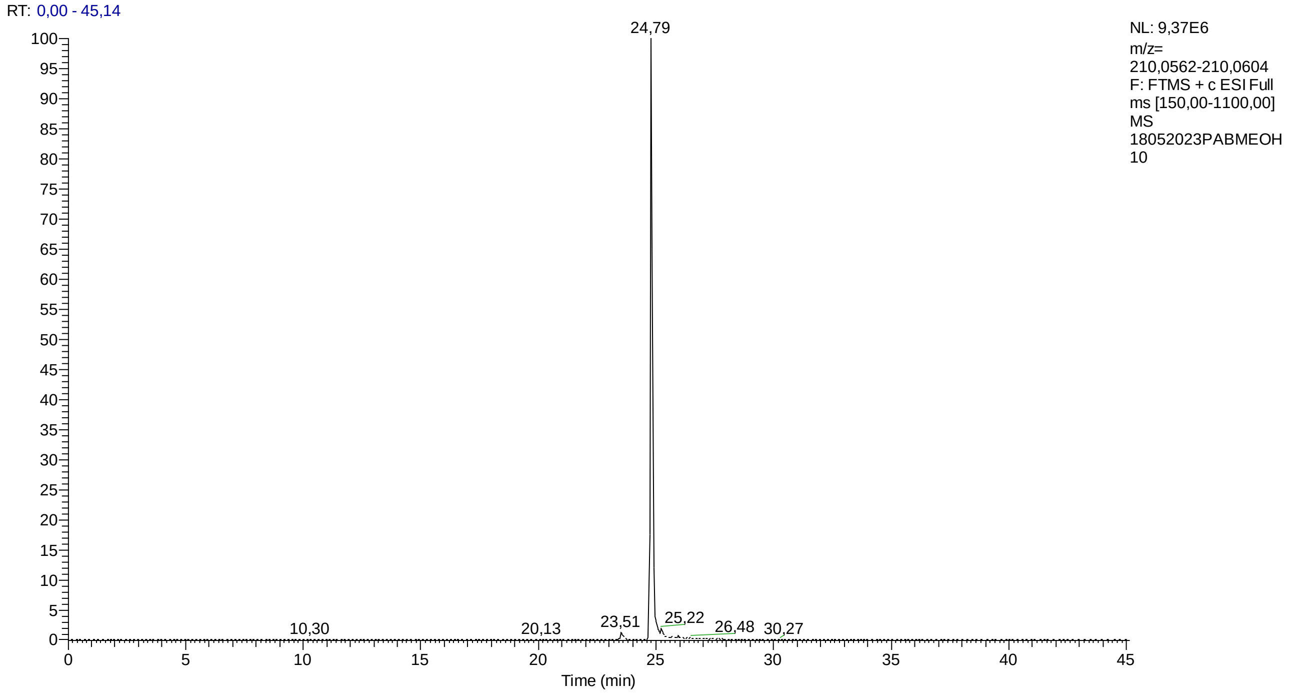

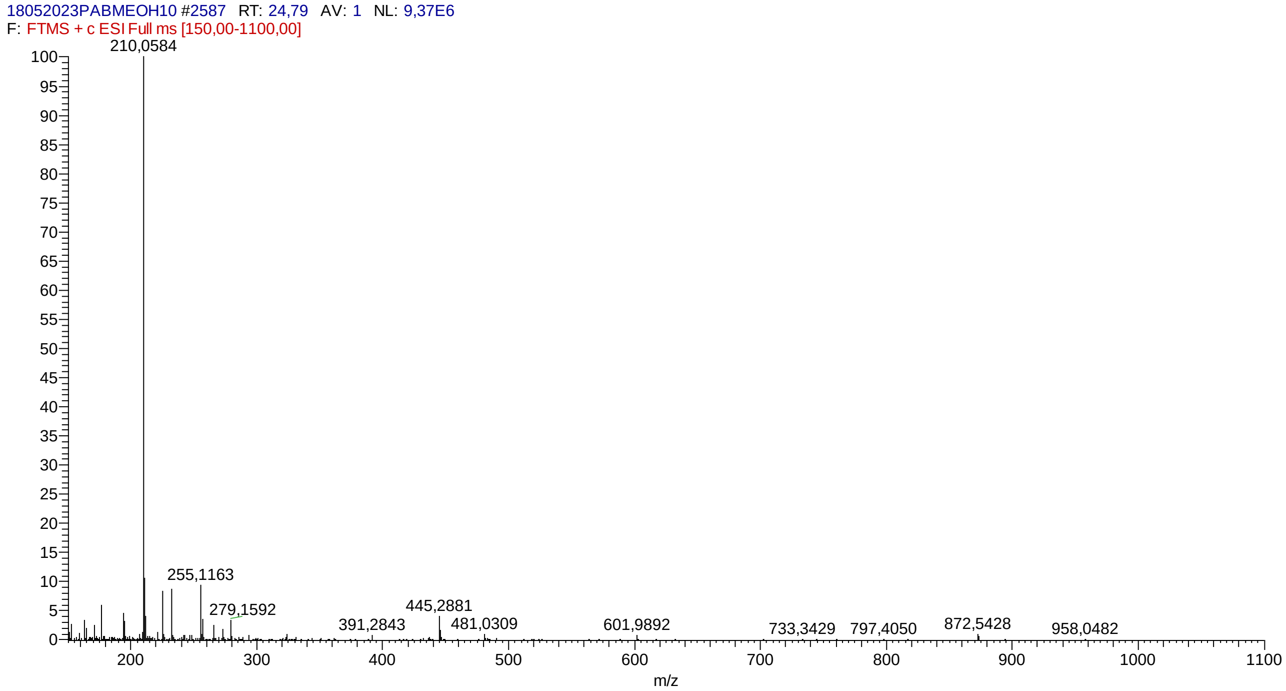

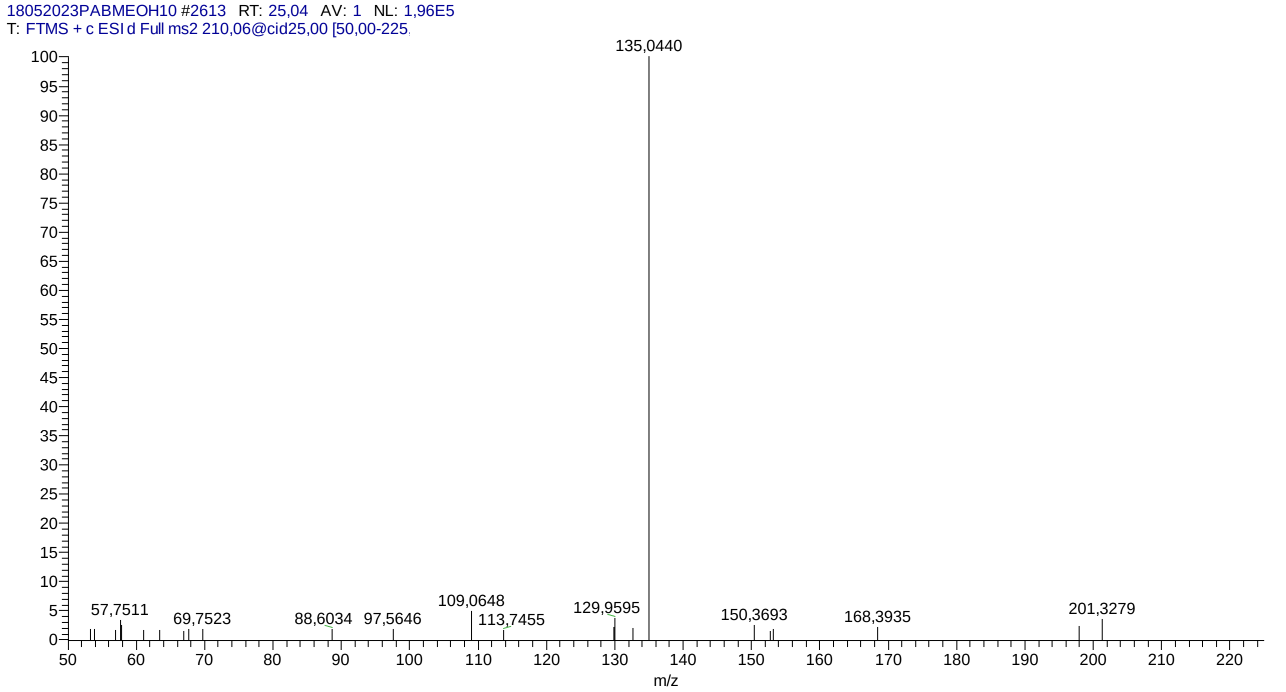

Figure S3. LC-HRMS analyses of **3** on an LTQ Orbitrap XL™ Hybrid FT Mass Spectrometer system: (**A**) Extracted ion Chromatogram (XIC) obtained by selecting the ion at *m/z* 210.0584, (**B**) relevant HRMS spectrum and (**C**) relevant HRMS^2 spectrum (Collision Induced Dissociation mode) obtained by using the ion at *m/z* 210.1 as precursor.

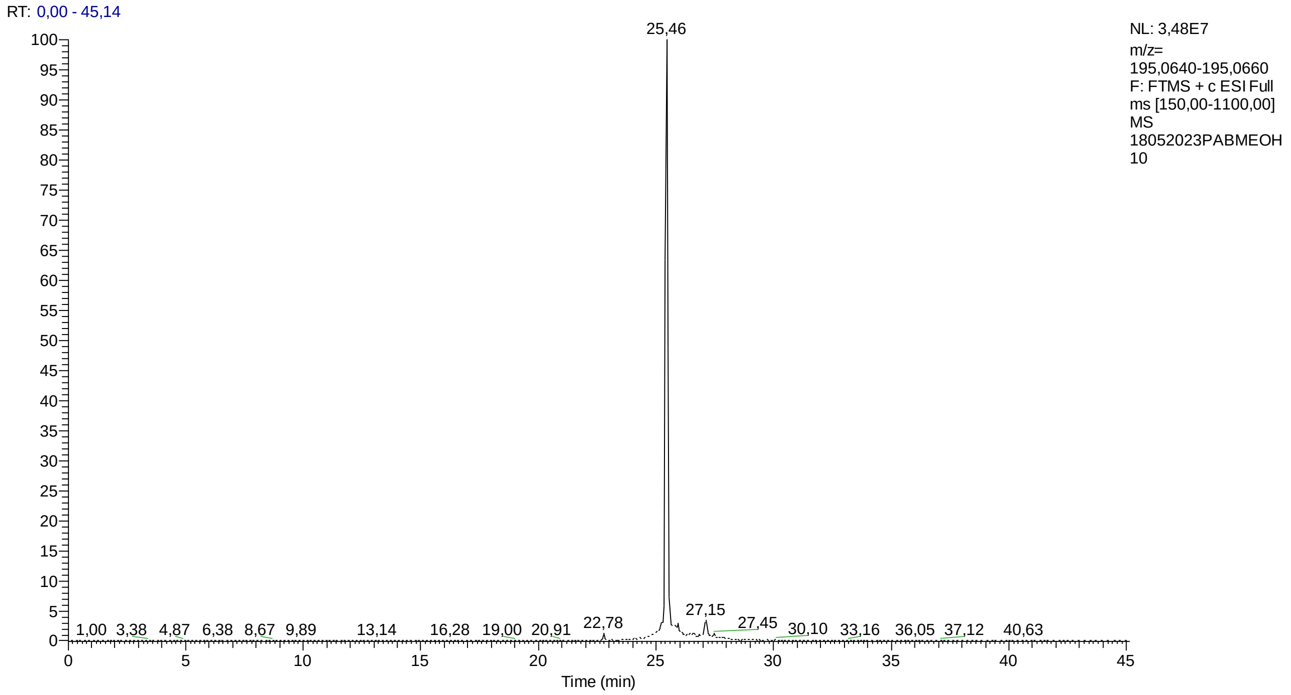
RT: 0,00 - 45,14
NL: 3,48E7
m/z=
195,0640-195,0660
F: FTMS + c ESI Full
ms [150,00-1100,00]
MS
18052023PABMEOH
10
25,46
1,00 3,38 4,87 6,38 8,67 9,89 13,14 16,28 19,00 20,91 22,78 27,15 27,45 30,10 33,16 36,05 37,12 40,63
Time (min)

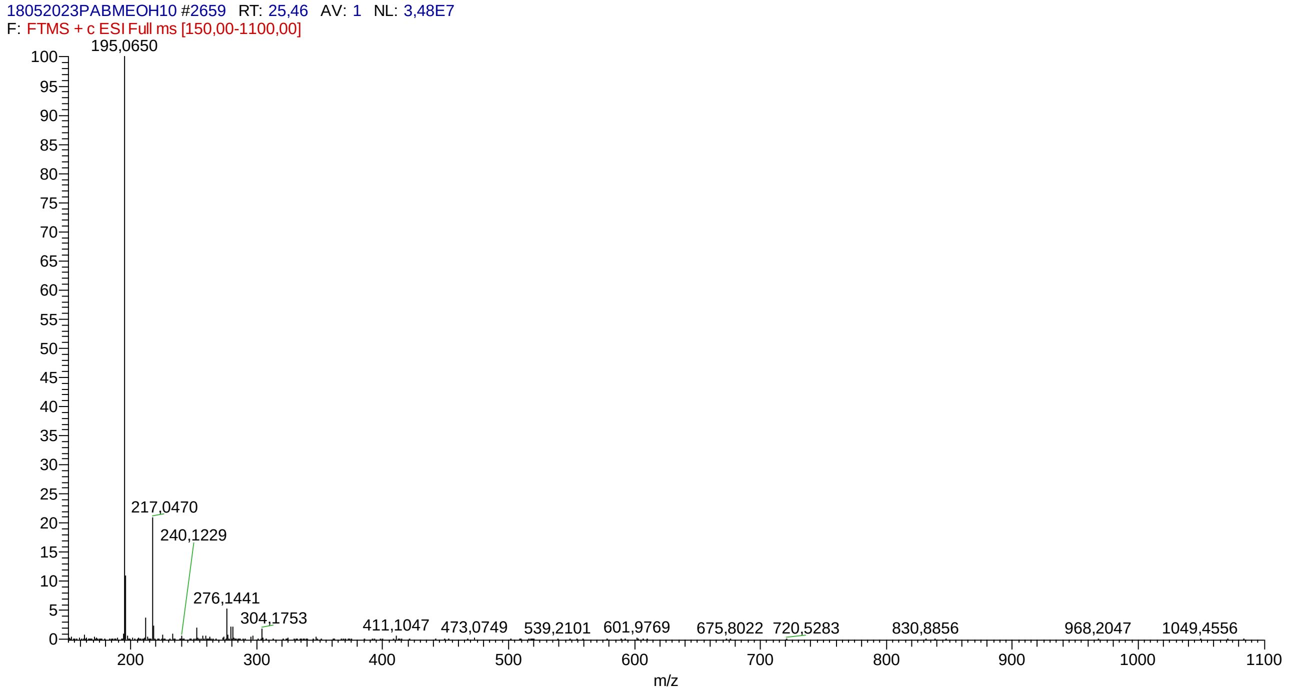
18052023PABMEOH10 #2659 RT: 25,46 AV: 1 NL: 3,48E7
F: FTMS + c ESI Full ms [150,00-1100,00]
195,0650
217,0470
240,1229
276,1441
304,1753
411,1047 473,0749 539,2101 601,9769 675,8022 720,5283 830,8856 968,2047 1049,4556
m/z

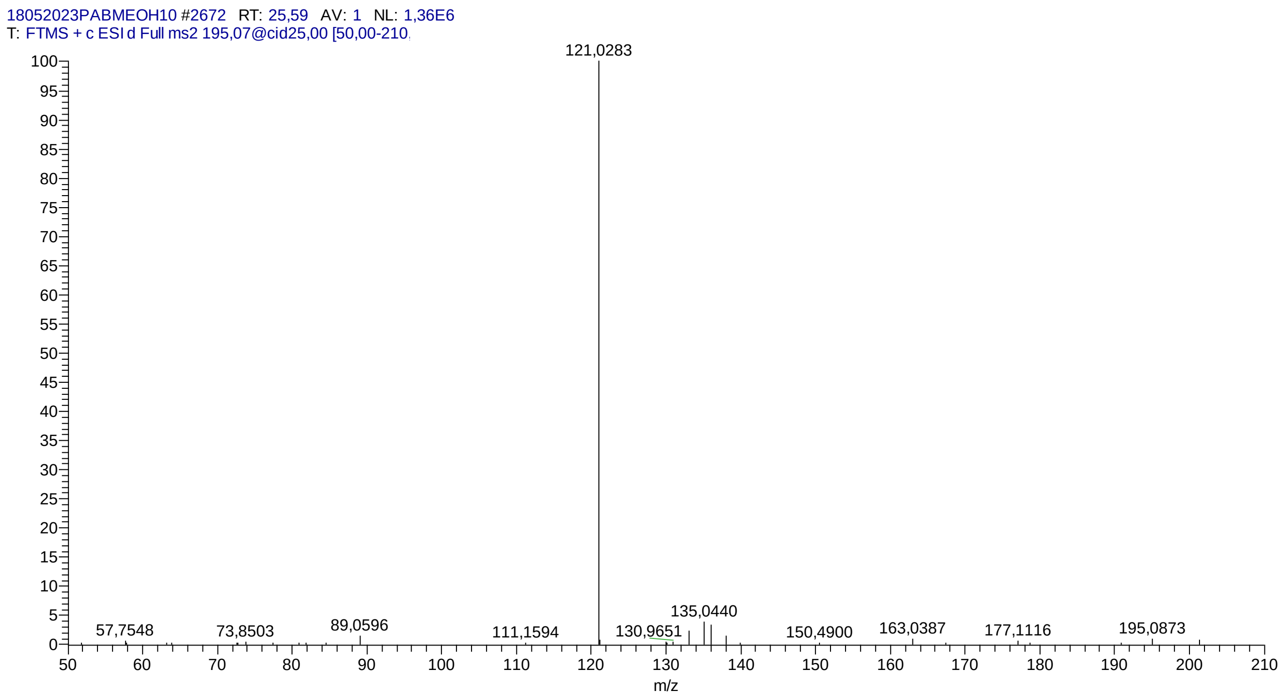

Figure S4. LC-HRMS analyses of **4** on an LTQ Orbitrap XL™ Hybrid FT Mass Spectrometer system: (**A**) Extracted ion Chromatogram (XIC) obtained by selecting the ion at *m/z* 195.0650, (**B**) relevant HRMS spectrum and (**C**) relevant HRMS[2] spectrum (Collision Induced Dissociation mode) obtained by using the ion at *m/z* 195.1 as precursor.

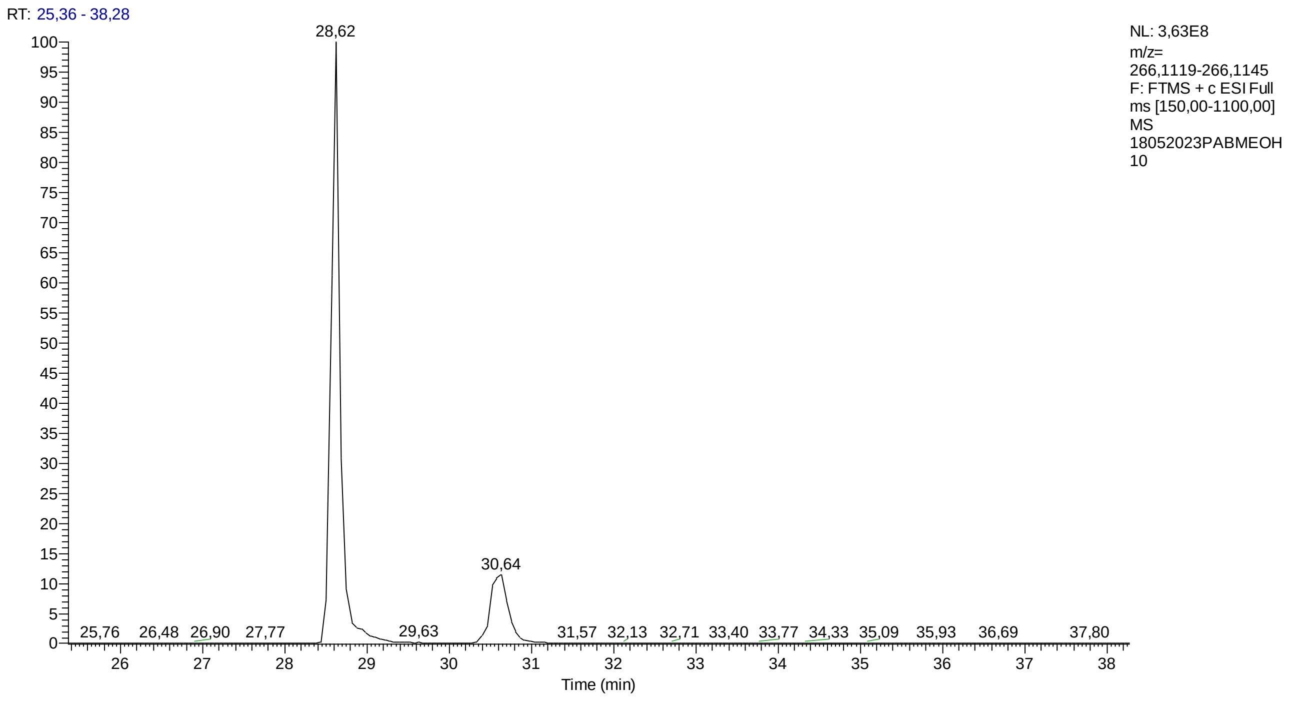

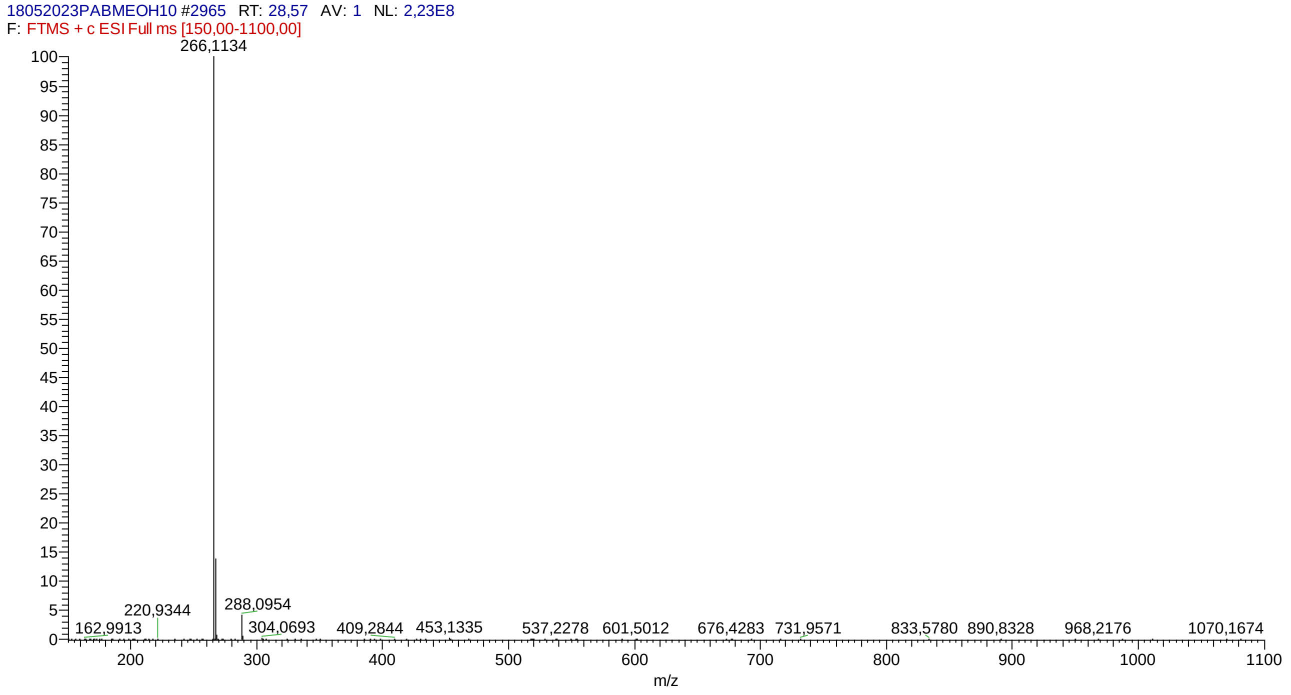
18052023PABMEOH10 #2965 RT: 28,57 AV: 1 NL: 2,23E8
F: FTMS + c ESI Full ms [150,00-1100,00]
266,1134
220,9344
288,0954
162,9913
304,0693
409,2844
453,1335
537,2278
601,5012
676,4283
731,9571
833,5780
890,8328
968,2176
1070,1674
m/z

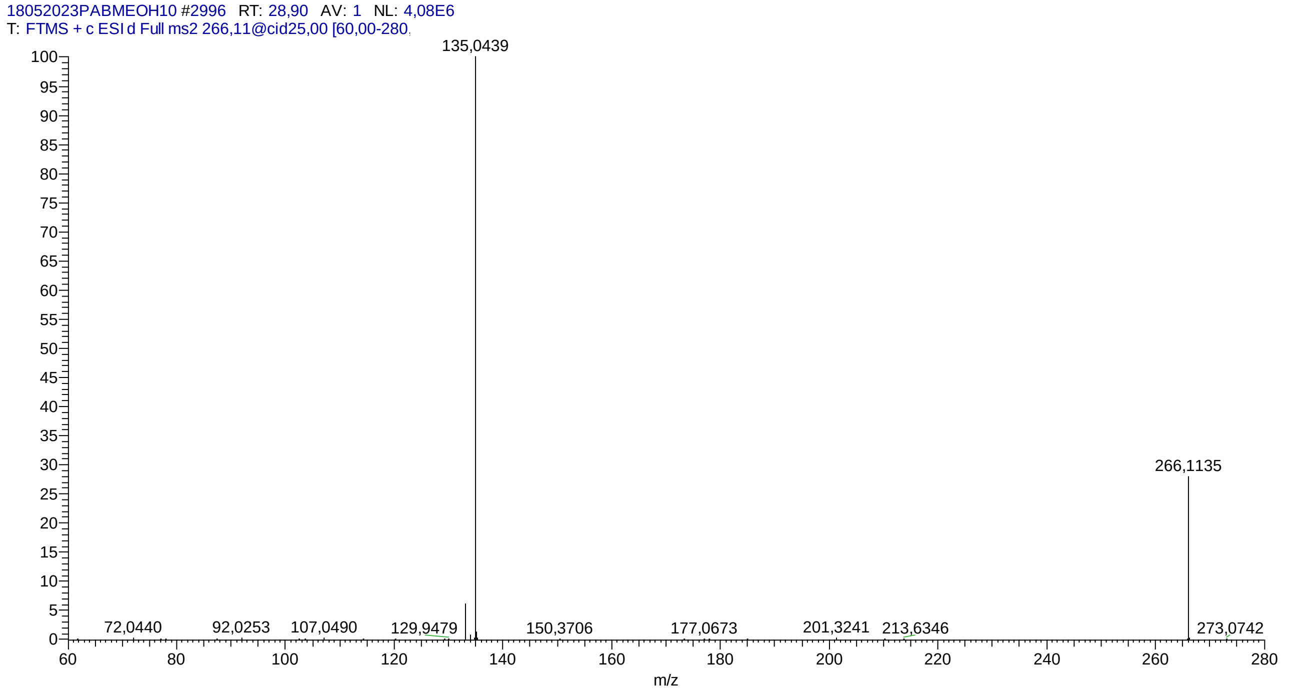
18052023PABMEOH10 #2996 RT: 28,90 AV: 1 NL: 4,08E6
T: FTMS + c ESI d Full ms2 266,11@cid25,00 [60,00-280,
135,0439
266,1135
72,0440
92,0253
107,0490
129,9479
150,3706
177,0673
201,3241
213,6346
273,0742
m/z

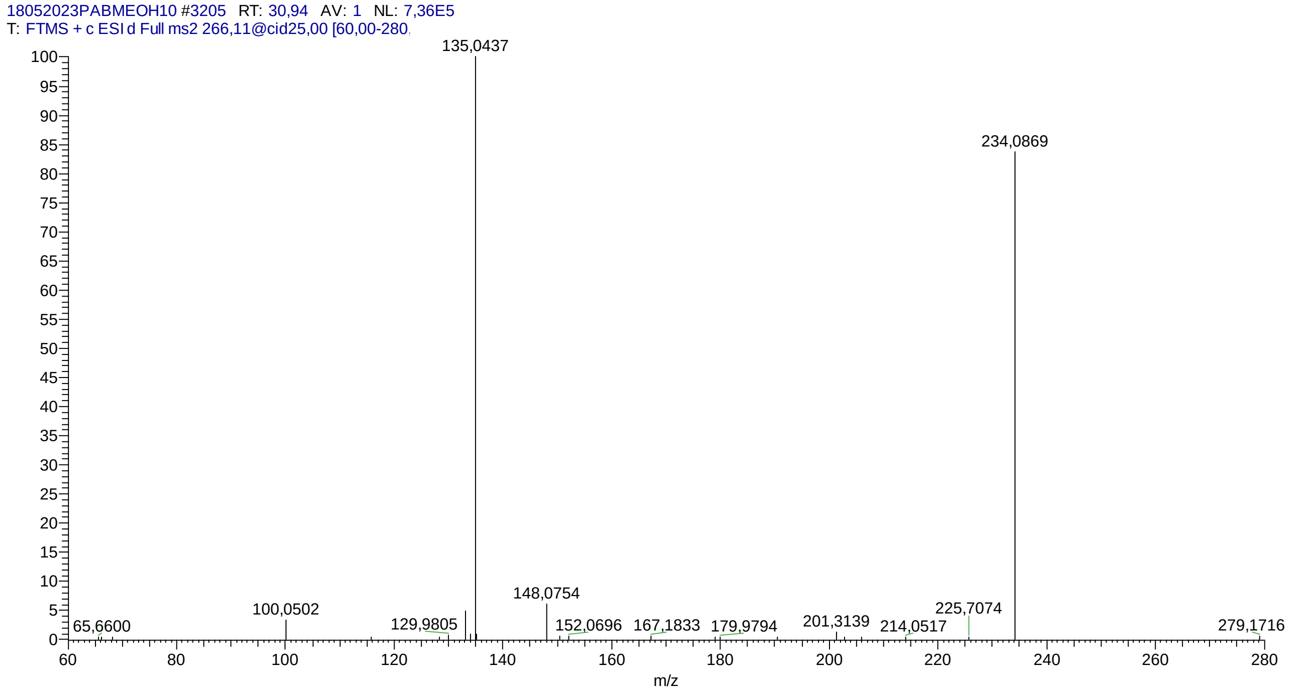

Figure S5. LC-HRMS analyses of **5** on an LTQ Orbitrap XL™ Hybrid FT Mass Spectrometer system: (**A**) Extracted ion Chromatogram (XIC) obtained by selecting the ion at *m/z* 266.1134, (**B**) relevant HRMS spectrum and (**C**) relevant HRMS² spectrum (Collision Induced Dissociation mode) obtained by using the ion at *m/z* 266.1 as precursor.

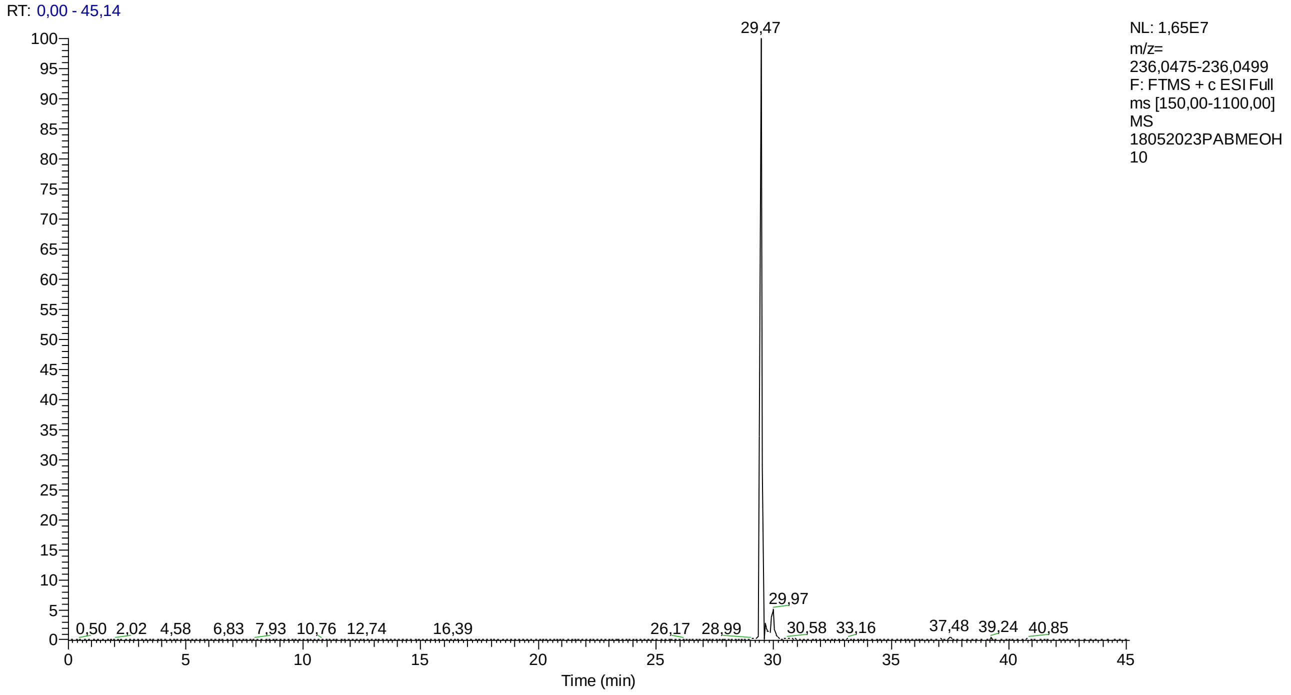
RT: 0,00 - 45,14
NL: 1,65E7
m/z= 236,0475-236,0499
F: FTMS + c ESI Full ms [150,00-1100,00]
MS 18052023PABMEOH10
29,47
29,97
0,50 2,02 4,58 6,83 7,93 10,76 12,74 16,39 26,17 28,99 30,58 33,16 37,48 39,24 40,85
Time (min)

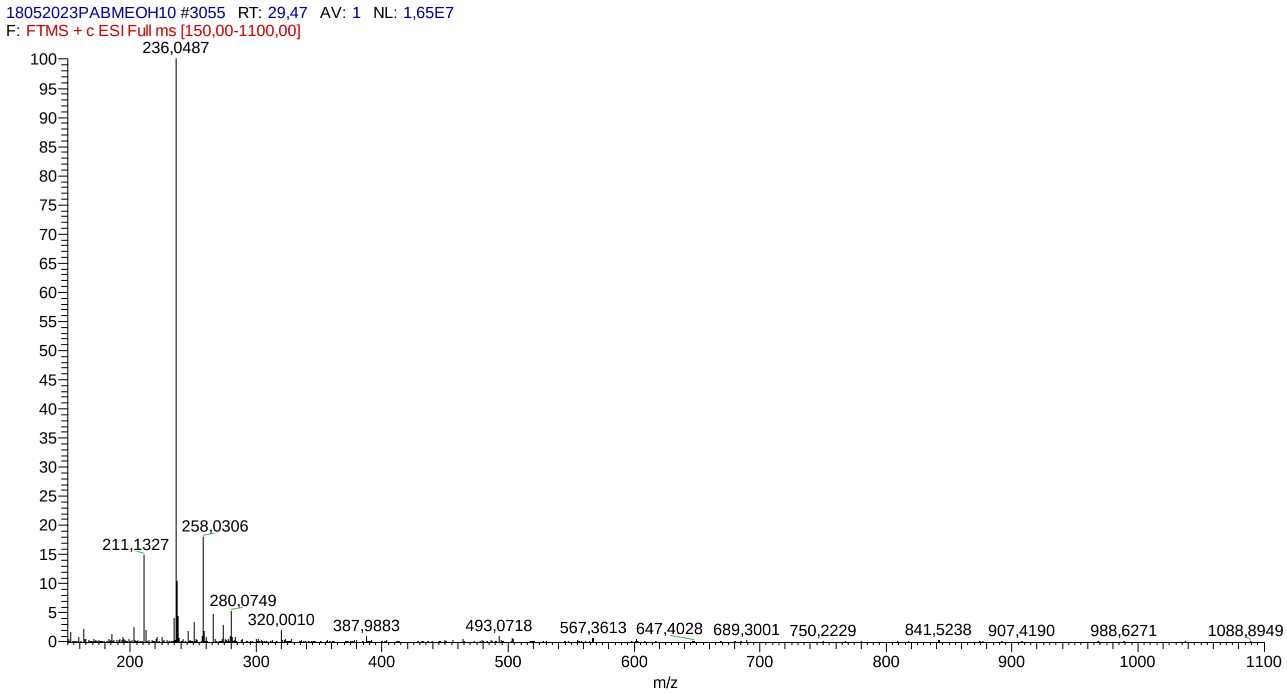
18052023PABMEOH10 #3055 RT: 29,47 AV: 1 NL: 1,65E7
F: FTMS + c ESI Full ms [150,00-1100,00]
236,0487
211,1327
258,0306
280,0749
320,0010
387,9883
493,0718
567,3613
647,4028
689,3001
750,2229
841,5238
907,4190
988,6271
1088,8949
m/z

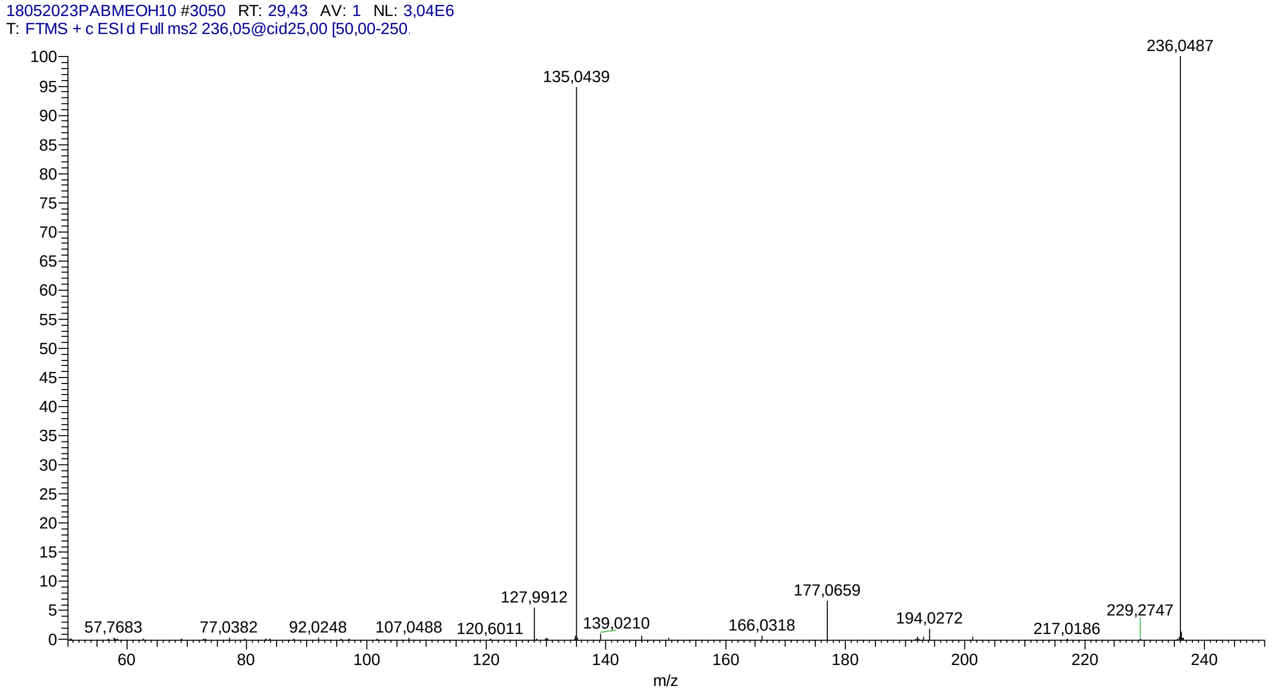

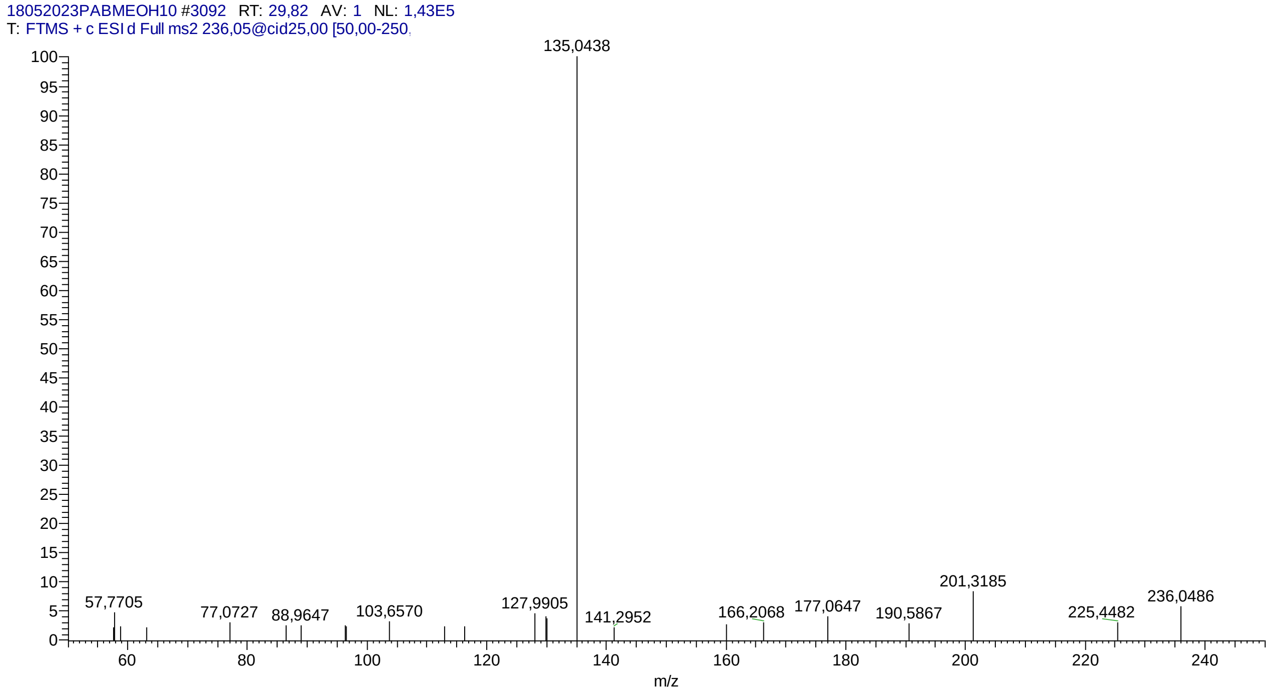

Figure S6. LC-HRMS analyses of **6** on an LTQ Orbitrap XL™ Hybrid FT Mass Spectrometer system: (**A**) Extracted ion Chromatogram (XIC) obtained by selecting the ion at *m/z* 236.0487, (**B**) relevant HRMS spectrum and (**C**) relevant HRMS[2] spectrum (Collision Induced Dissociation mode) obtained by using the ion at *m/z* 236.0 as precursor.

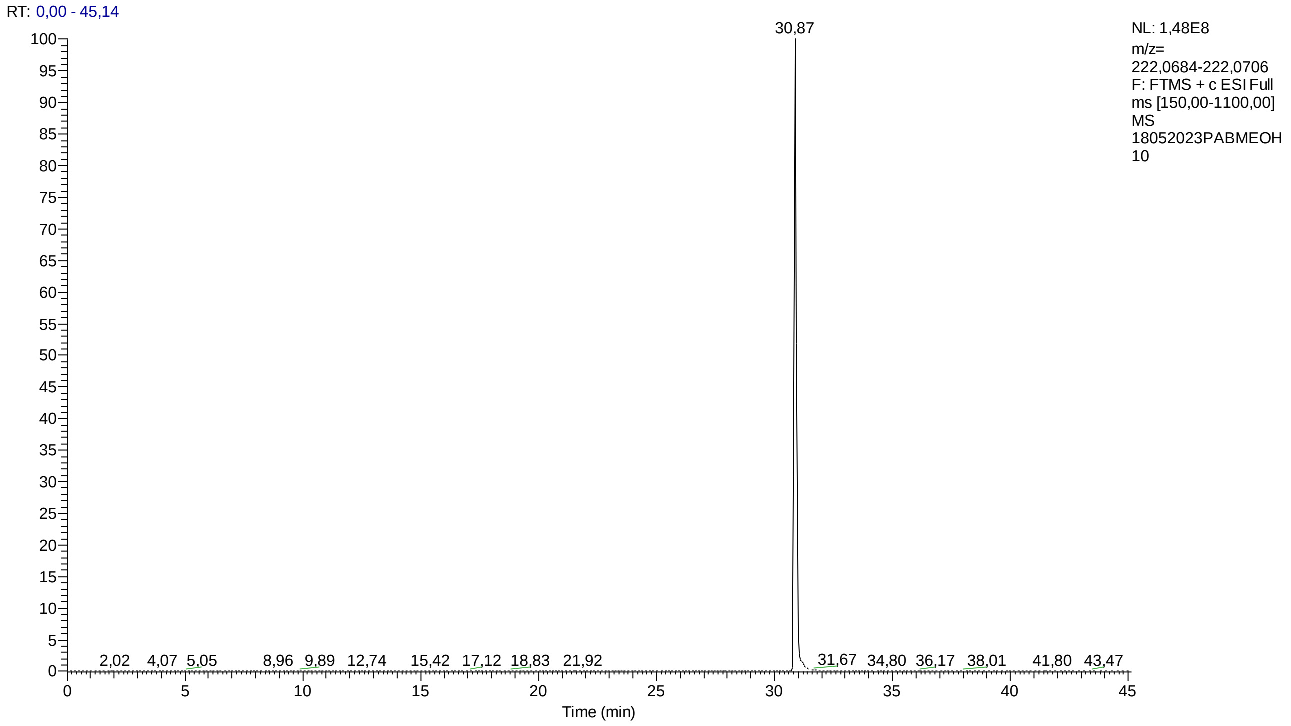

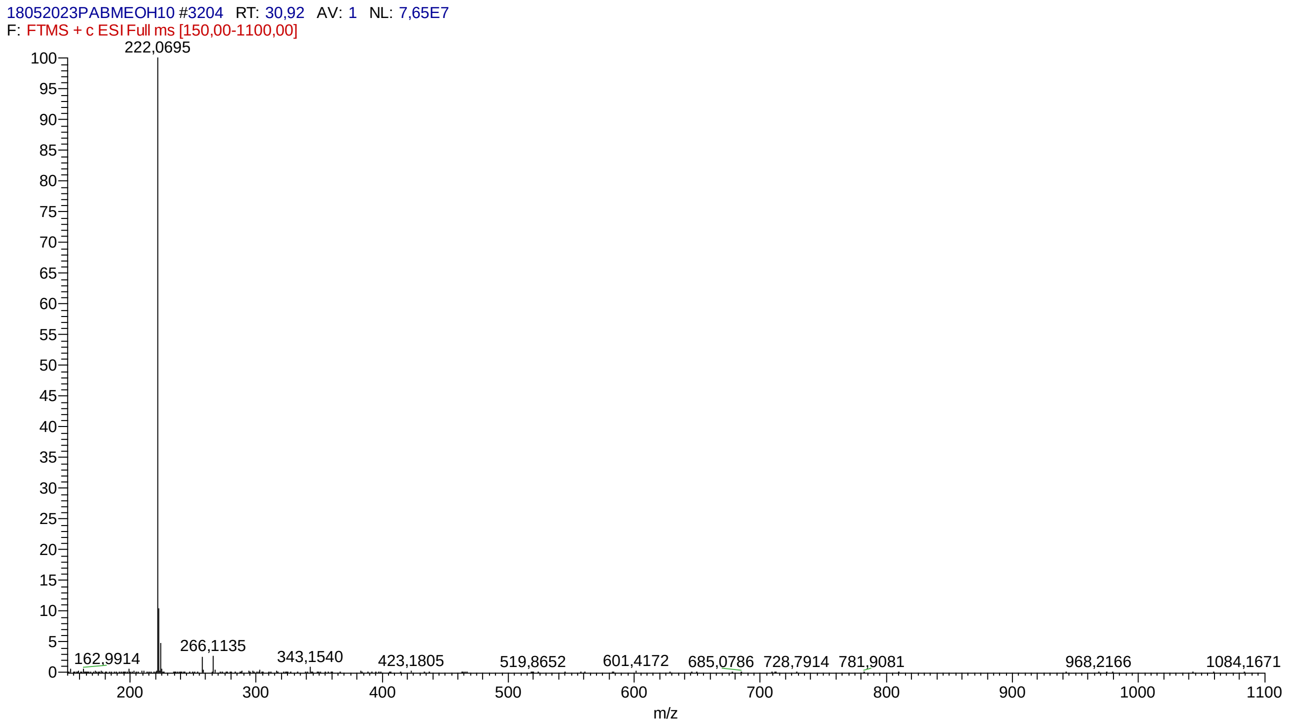

Figure S7. LC-HRMS analyses of **7** on an LTQ Orbitrap XL™ Hybrid FT Mass Spectrometer system: (**A**) Extracted ion Chromatogram (XIC) obtained by selecting the ion at *m/z* 222.0695, (**B**) relevant HRMS spectrum.

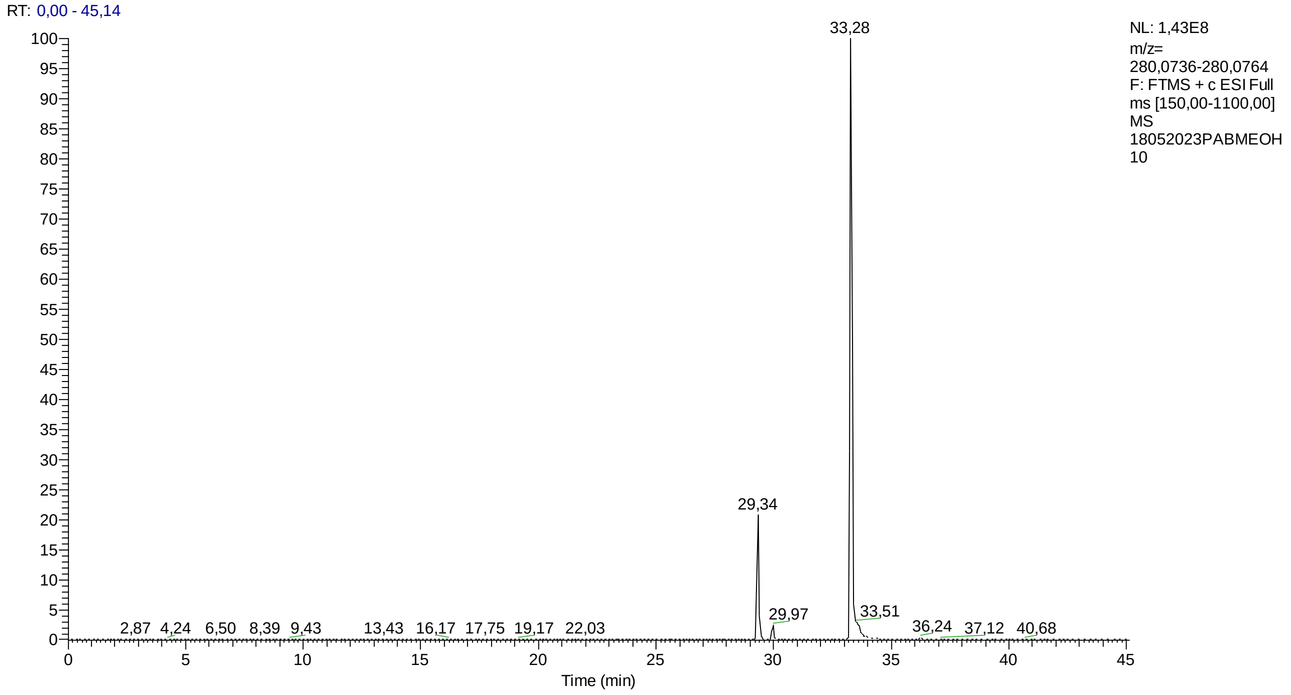
RT: 0,00 - 45,14
NL: 1,43E8
m/z= 280,0736-280,0764
F: FTMS + c ESI Full ms [150,00-1100,00]
MS 18052023PABMEOH10
33,28
29,34
29,97
33,51
2,87 4,24 6,50 8,39 9,43 13,43 16,17 17,75 19,17 22,03
36,24 37,12 40,68
Time (min)

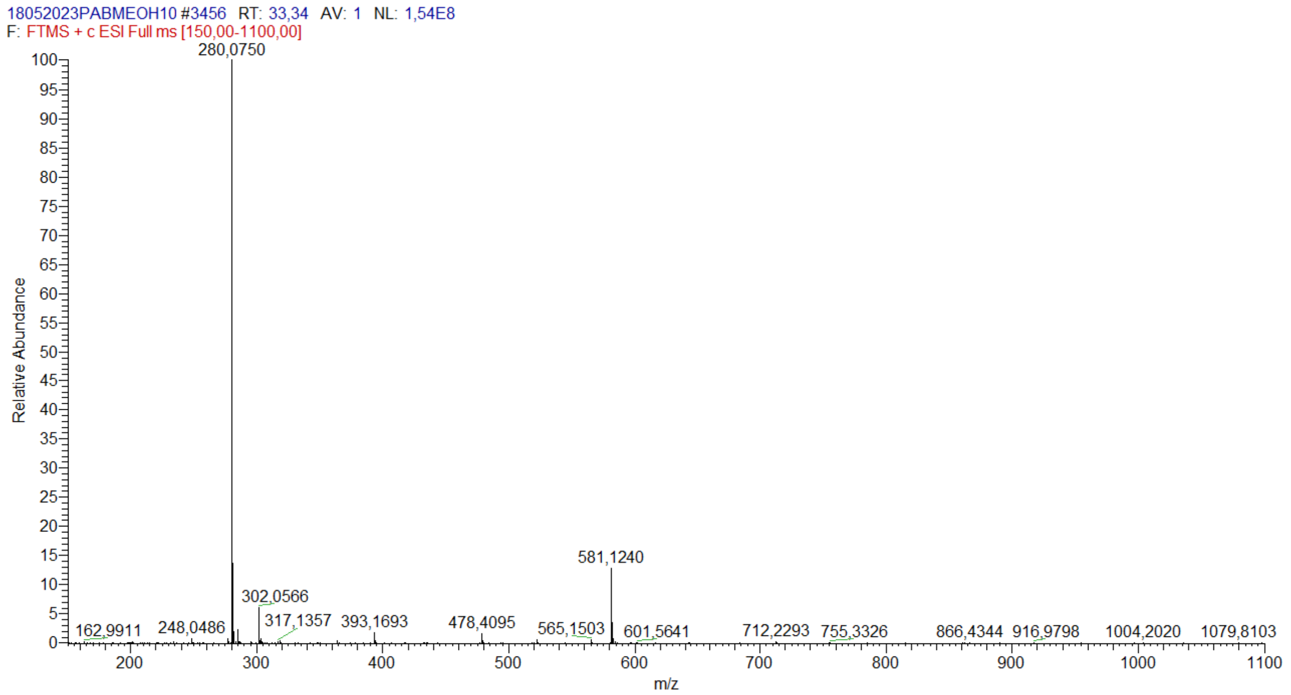
18052023PABMEOH10 #3456 RT: 33,34 AV: 1 NL: 1,54E8
F: FTMS + c ESI Full ms [150,00-1100,00]
280,0750
581,1240
302,0566
162,9911 248,0486 317,1357 393,1693 478,4095 565,1503 601,5641 712,2293 755,3326 866,4344 916,9798 1004,2020 1079,8103
Relative Abundance
m/z

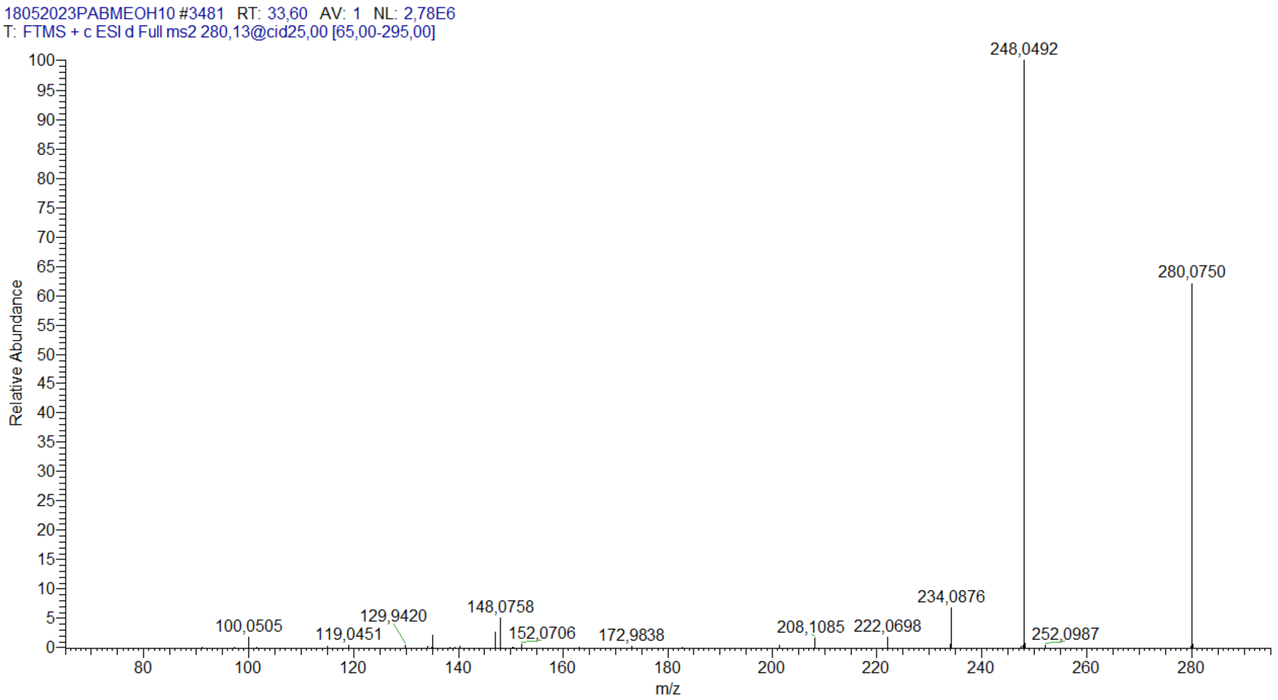

Figure S8. LC-HRMS analyses of **8** on an LTQ Orbitrap XL™ Hybrid FT Mass Spectrometer system: (**A**) Extracted ion Chromatogram (XIC) obtained by selecting the ion at *m/z* 280.0750, (**B**) relevant HRMS spectrum and (**C**) relevant HRMS^2 spectrum (Collision Induced Dissociation mode) obtained by using the ion at *m/z* 280.1 as precursor.

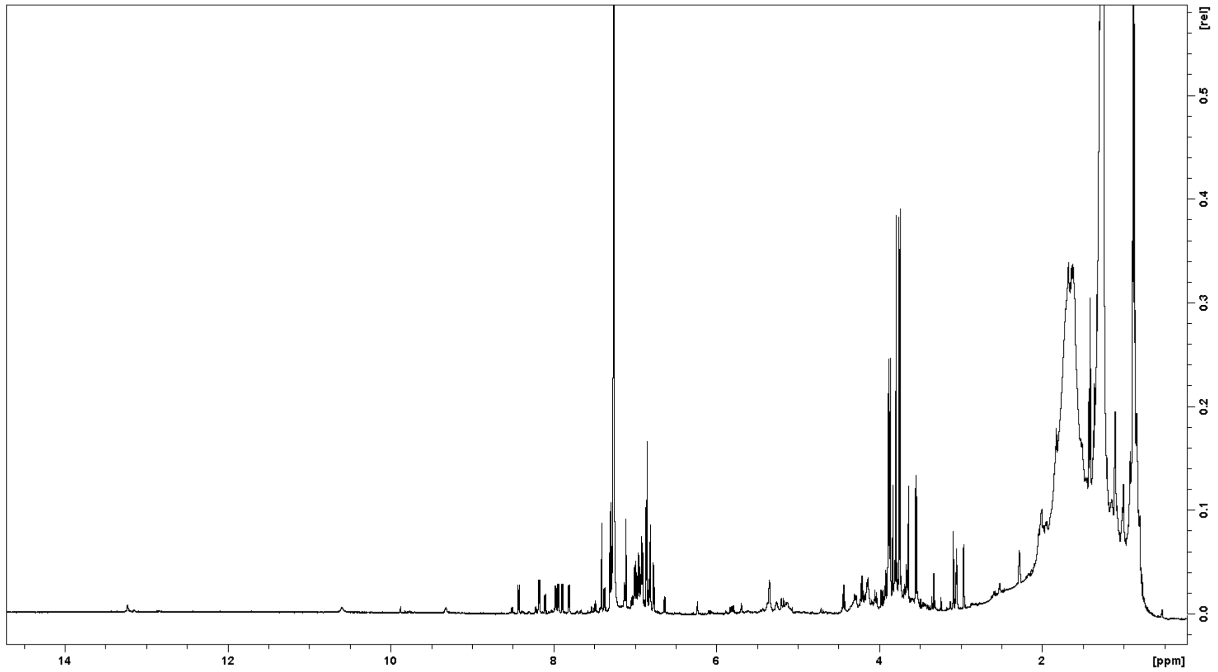

Figure S9. ^{1}H NMR spectrum (700 MHz) in $CDCl_3$ of PAB2.

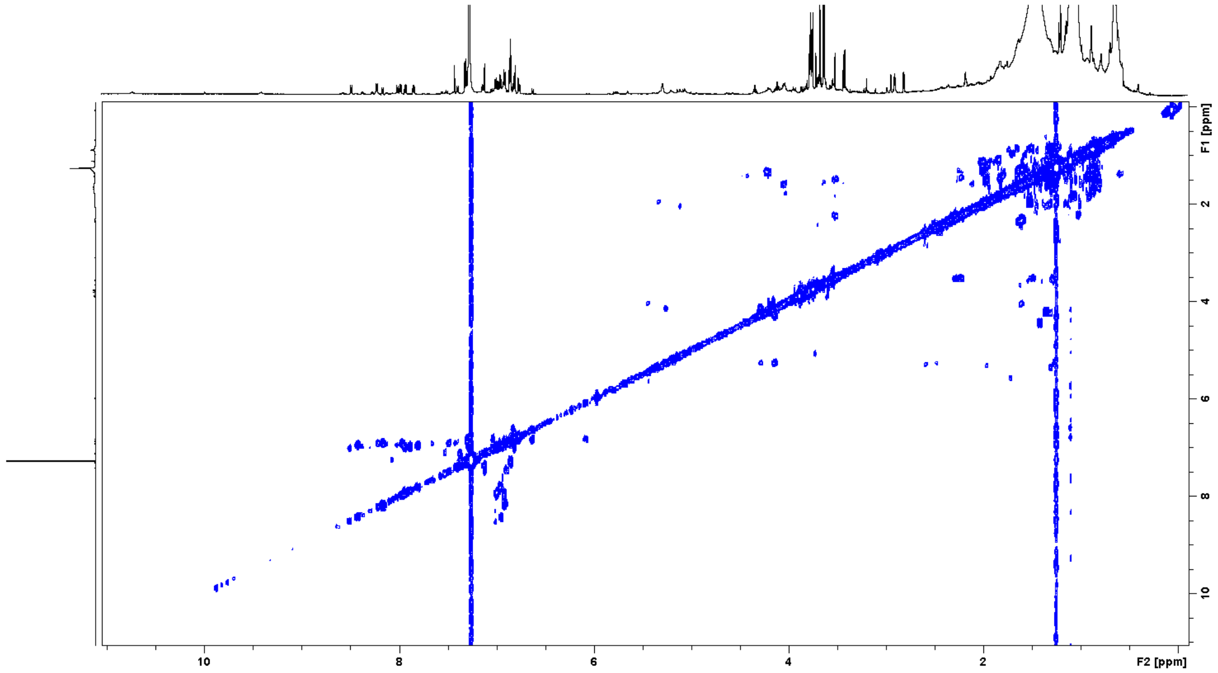

Figure S10. COSY spectrum in $CDCl_3$ of PAB2.

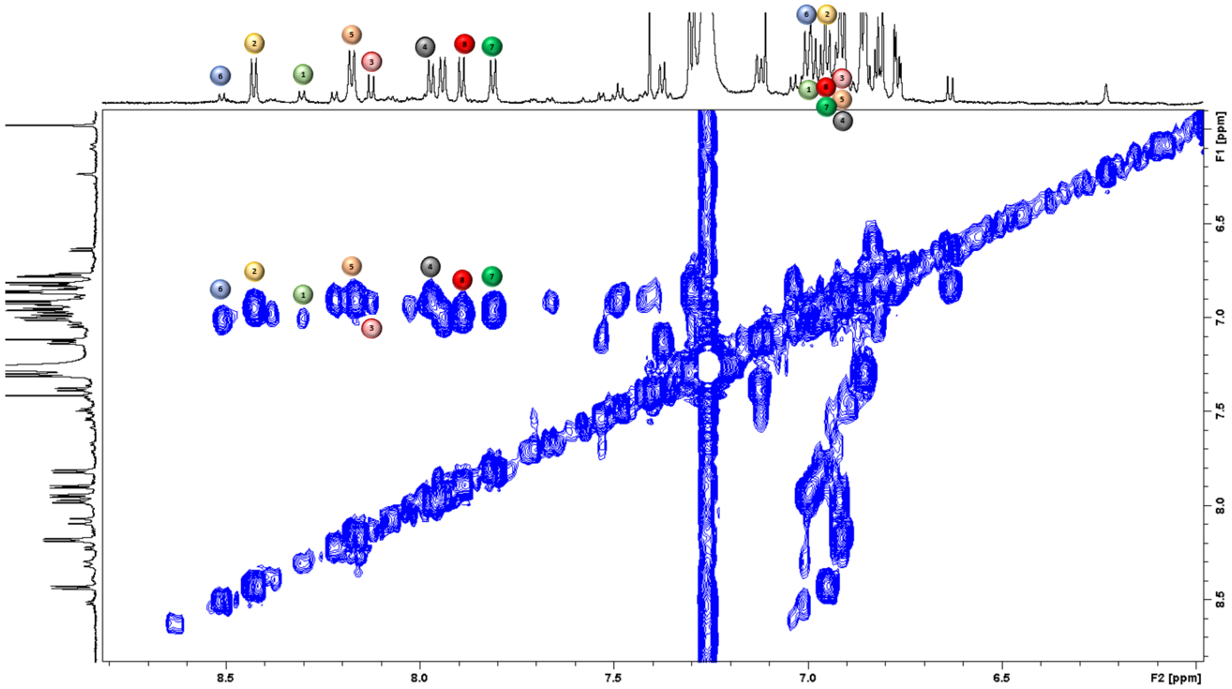

Figure S11. Downfield region enlargement of COSY spectrum in $CDCl_3$ of PAB2.

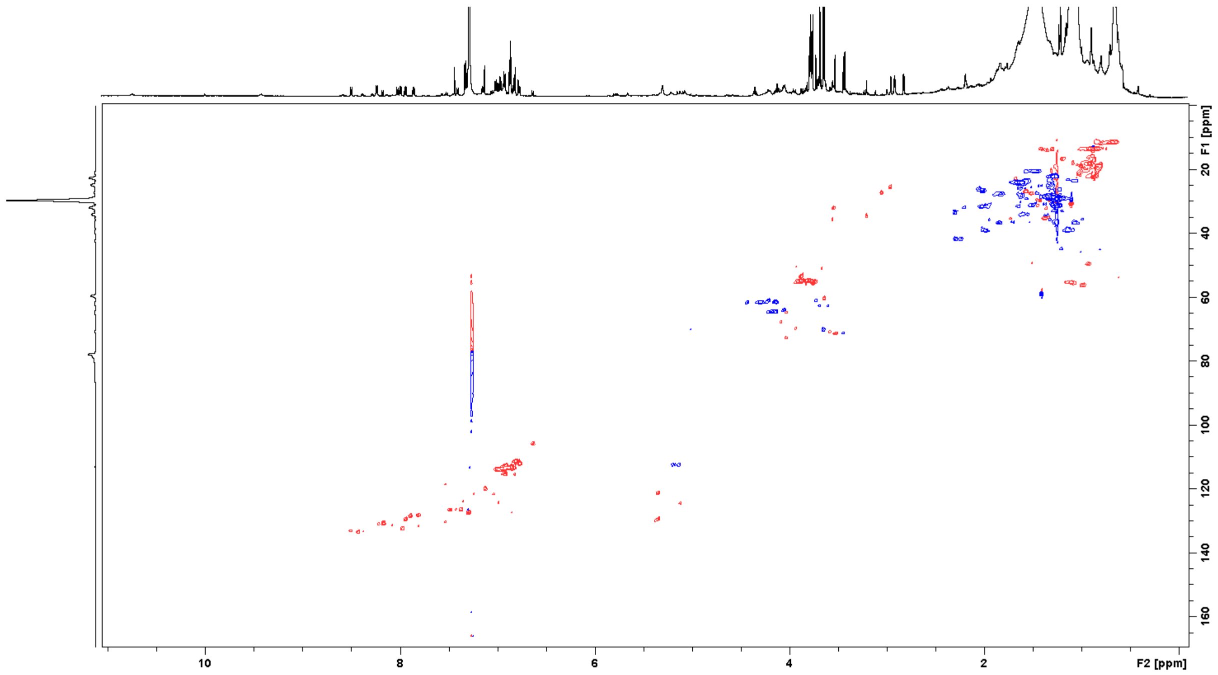

Figure S12. HSQC spectrum in $CDCl_3$ of PAB2.

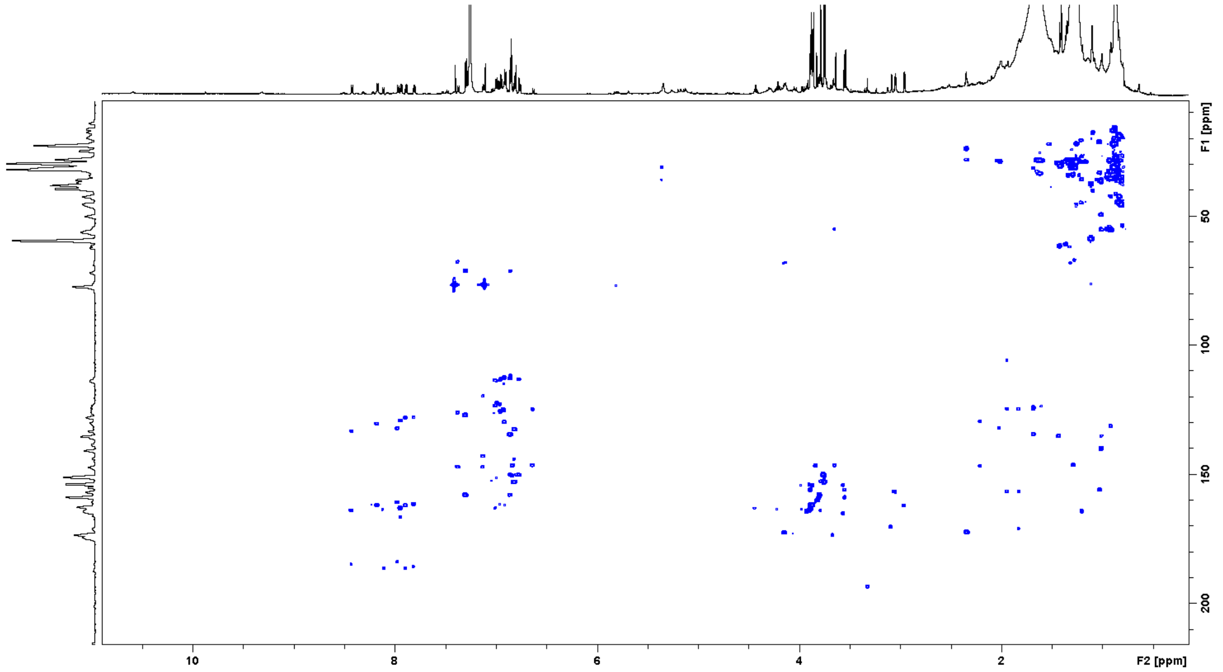

Figure S13. HMBC spectrum in $CDCl_3$ of PAB2.

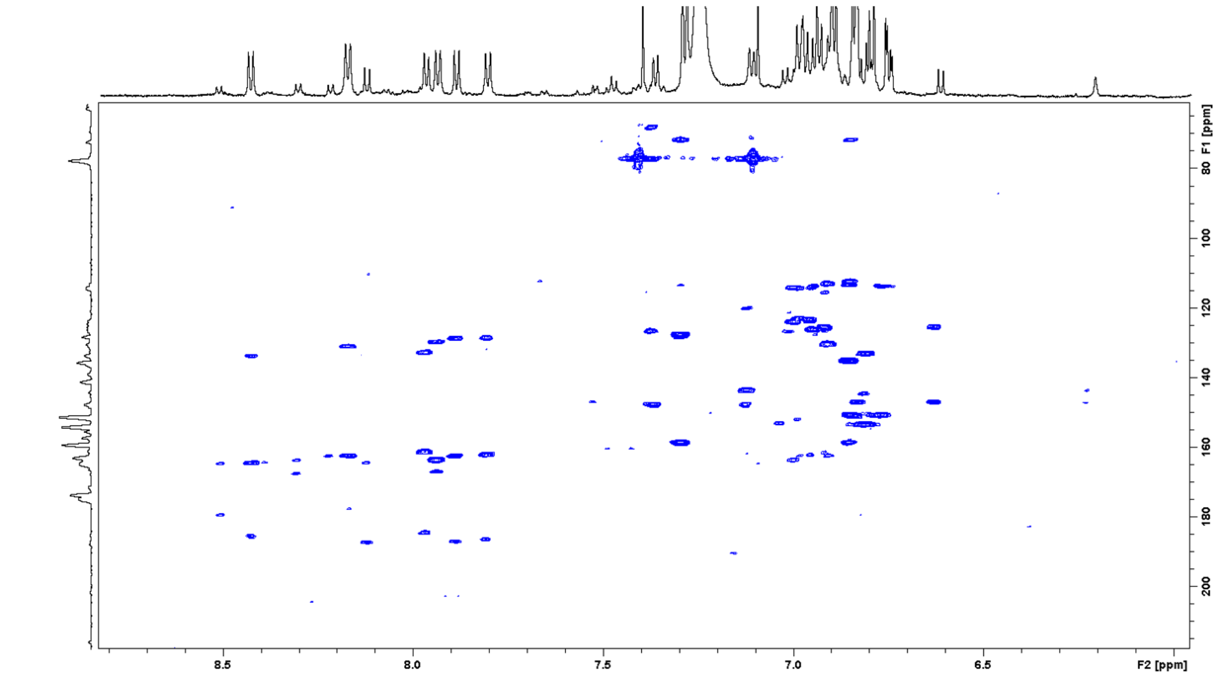

Figure S14. Downfield region enlargement of HMBC spectrum in $CDCl_3$ of PAB2.
